# Metagenomic analysis of the interaction between the gut microbiota and colorectal cancer: a paired-sample study based on the GMrepo database

**DOI:** 10.1186/s13099-022-00527-8

**Published:** 2022-12-23

**Authors:** Han Chen, Jianhua Jiao, Min Wei, Xingzhou Jiang, Ruoyun Yang, Xin Yu, Guoxin Zhang, Xiaoying Zhou

**Affiliations:** 1 Department of Gastroenterology, The First Affiliated Hospital of Nanjing Medical, University300# Guangzhou Road, Nanjing, 210029 People’s Republic of China; 2 The First Clinical Medical College, Nanjing Medical University, Nanjing, China; 3 Department of Gastroenterology, Nanjing Jiangning Hospital, Nanjing, China

**Keywords:** Colorectal cancer, Gut microbiota, Metagenomic analysis, Matched case‒control study, GMrepo Database

## Abstract

**Background:**

Previous evidence has shown that the gut microbiota plays a role in the development and progression of colorectal cancer (CRC). This study aimed to provide quantitative analysis and visualization of the interaction between the gut microbiota and CRC in order to establish a more precise microbiota panel for CRC diagnosis.

**Method:**

A paired-sample study was designed by retrieving original metagenomic data from the GMrepo database. The differences in the distribution of the gut microbiota between CRCs and controls were analysed at the species level. A co-occurrence network was established, and the microbial interactions with environmental factors were assessed. Random forest models were used to determine significant biomarkers for differentiating CRC and control samples.

**Results:**

A total of 709 metagenomic samples from 6 projects were identified. After matching, 86 CRC patients and 86 matched healthy controls from six countries were enrolled. A total of 484 microbial species and 166 related genera were analysed. In addition to previously recognized associations between *Fusobacterium nucleatum* and species belonging to the genera *Peptostreptococcus*, *Porphyromonas*, and *Prevotella* and CRC, we found new associations with the novel species of *Parvimonas micra* and *Collinsella tanakaei*. In CRC patients, *Bacteroides uniformis* and *Collinsella tanakaei* were positively correlated with age, whereas *Dorea longicatena*, *Adlercreutzia equolifaciens*, and *Eubacterium hallii* had positive associations with body mass index (BMI). Finally, a random forest model was established by integrating different numbers of species with the highest model-building importance and lowest inner subcategory bias. The median value of the area under the receiver operating characteristic curve (AUC) was 0.812 in the training cohort and 0.790 in the validation set.

**Conclusions:**

Our study provides a novel bioinformatics approach for investigating the interaction between the gut microbiota and CRC using an online free database. The identification of key species and their associated genes should be further emphasized to determine the relative causality of microbial organisms in the development of CRC.

**Supplementary Information:**

The online version contains supplementary material available at 10.1186/s13099-022-00527-8.

## Introduction

Colorectal cancer (CRC) ranks third in terms of both new incidence and cancer-related mortality according to the latest data of cancer statistics for the United States [1]. The pathogenesis of CRC is closely associated with complex interactions between heritable factors and environmental lifestyle factors [2]. Although genome-wide association studies have already identified several genes involved in CRC progression, only 5–7% of CRCs can be explained by a well-defined gene-regulated sequence [3]. Therefore, the potential function of environmental factors should be further studied to determine the underlying mechanisms that may trigger sporadic colorectal carcinogenesis.

The gut microbiota is reported to participate in CRC development, progression, and even the individual’s therapeutic response to anticancer medications [4]. The gut microbiota has been increasingly reported as a possible mechanistic link between CRC and environmental factors [5, 6]. Several studies have demonstrated that microbial dysbiosis may contribute to CRC pathogenesis, possibly due to constant crosstalk between intestinal epithelial cells and luminal microorganisms [7, 8]. Some recent studies [9–11] have analysed the differences in the gut microbiota in CRC and healthy controls using faecal sequencing techniques. Some species, such as *Bacteroides fragilis*, *Escherichia coli*, *Fusobacterium nucleatum*, and *Peptostreptococcus species,* have been reported to be potential microbiologic markers for improving CRC diagnoses. Since a difference in the abundance of certain gut bacteria has been found in CRC patients, these combinations of bacteria can be regarded as novel diagnostic panels to screen for CRC.

With the rapid development of bioinformatics analysis, several databases have been developed to guide scientific research on the gut microbiota. The GMrepo database is an easily accessed and well-organized electronic database that facilitates the accessibility of the rapidly growing amount of human metagenomic data. GMrepo provides microbiota data from different areas and then classifies these data according to different phenotypes and all possible related meta-data, such as age, sex, country, and body mass index (BMI) [12].

Here, we designed a metagenomic analysis method with paired samples to identify promising microorganism-specific biomarkers that contribute to CRC tumorigenesis based on the metagenomic data in the GMrepo database. As male sex, increased age, and excessive body weight have all been shown to be independently related to increased risks of CRC [13–15], we eliminated the interference of these confounding factors to focus on identifying more reliable microbial factors that trigger cancer progression by enrolling samples with matched sex, age, region, and BMI. Thus, the present study established more precise microorganism panels for CRC diagnosis.

## Methods

### Database

The metagenomic analysis was based on information from the GMrepo database, which contains more than 58,000 human gut samples/runs (including both metagenomes and amplicons) relating to 92 disease phenotypes [12]. All included patients underwent whole metagenome sequencing, and taxonomic profiles were generated.

### Study design and data collection

This study enrolled patients with colorectal neoplasms (Medical Subject Headings (MeSH) Unique ID: D015179) and healthy controls (MeSH Unique ID: D006262). We included patients (1) diagnosed with colorectal neoplasms; (2) with a positive quality control (QC) status in the database; and (3) with associated metagenomic sequence data that was available. The exclusion criteria were as follows: (1) a recent history of antibiotic use; (2) amplicon data; and (3) missing information on sex, age, or BMI. After applying similar criteria, controls without colorectal neoplasms were matched at a 1:1 ratio to cases by age (± 3 years), sex, BMI (± 0.5 kg/m^2^), and region. Each data point of relative abundance from the samples was collected and finally integrated into an operational taxonomic unit (OTU) abundance table. The NCBI taxonomy database was utilized to classify organisms at different levels (Kingdom, Phylum, Class, Order, Family, Genus, and Species). The taxonomic composition of each sample was then integrated into a final taxonomy classification table. MedCalc (Version 20.100–64-bit) software was applied for sample size calculation using the area under the ROC curve (AUC) at the 0.05 α-level and for the 0.1 β-level (power is 90%); thus, the expected AUC was 0.8, and the null hypothesis value was set to 0.6. The ratio of the sample sizes in the negative/positive groups was 1.0 due to the paired design. The minimum sample size required for each group was 35.

### Statistical analysis

Statistical analysis was performed using SPSS Statistics for Windows, Version 25.0 (IBM Corporation, Armonk, NY), and R software (version 4.1.0). Normality tests involved Shapiro‒Wilk and Kolmogorov‒Smirnov tests. Data with a normal distribution were considered if the p-value was less than 0.05, and these data were presented as the mean and standard deviation. Data with a nonnormal distribution were presented as the median with an interquartile range (Q). For comparisons, the paired *t-*test (2-tailed) was applied for data with a normal distribution, while the Wilcoxon signed-rank test was performed for data with a nonnormal distribution. Wilcoxon Mann‒Whitney tests were performed for independent data with nonnormal distributions. Either Pearson’s chi-square test or Fisher’s exact test was applied to compare categorical variables. The function module of “case control matching” in SPSS was applied to reach a 1:1 ratio of case‒control matching. The “maps”, “scatterpie”, and “ggplot2” packages in R software were utilized to generate the world map.

### Analysis of alpha and beta diversity

Alpha diversity was evaluated by the Shannon index, Pielou evenness, Simpson index, and Equitability evenness using the vegan package in R software (version 4.1.0, http://www.R-project.org/). The difference in alpha diversity was calculated by the Wilcoxon signed-rank test. Beta diversity was presented by unconstrained principal coordinate analysis (PCoA) scatter plots by calculating Bray‒Curtis distances. Permutational multivariate analysis of variance (PERMANOVA) was then used to determine the differences between different phenotypes.

### Analysis of microbiome distribution differences

The Wilcoxon-signed rank test was applied to evaluate the microbiome distribution differences between the CRC and control cohorts. The packages “ggrepel” and “ggplot2” were used to generate a volcano plot. A Venn diagram was used to identify species exclusively present in the CRC or control groups. An UpSet plot was generated using the UpSetR package to identify unique and common OTUs. Clustering analysis after standardization of data by the Z score was performed in order to determine the different components in microbial species in the CRC and control groups using the pheatmap package [16]. The clustering analysis, Venn diagrams, and species distribution diagrams for different subgroups were generated using Wekemo BioinCloud (https://www.bioincloud.tech). To account for multiple testing, two-sided p-values were adjusted according to the false discovery rate (FDR) method. An association was considered to be statistically significant if its corresponding adjusted p-value was below 0.05, corresponding to an FDR of 5%.

### Identification of microbial biomarkers for CRC

Finally, a random forest model [19] was built. Significant microorganisms were incorporated into a panel for classifying CRC, and receiver operating characteristic (ROC) curve analysis was used to determine model performance using the “pROC” package [20]. The predictive performance was optimized by selecting species that displayed the best discriminatory power.

### Microbial interactions with environmental factors

We applied both the Spearman correlation and random forest algorithm. The Spearman correlation test was used to estimate the correlation between environmental factors and the gut microbiota. Additionally, the random Forest package was applied to identify age/BMI-discriminatory bacterial taxa lists [17]. The relative abundance of species was then regressed using default parameters, and the top 12 ranked species (with high values of the increased node impurity indexes (IncNodePurity)) were used to map the developmental spectrum of the gut microbiota in CRC groups. To further investigate the microbiotas association with age or BMI, we performed subgroup analysis as follows: ages were classified into three subgroups: age < 60 years, 60 ≤ age < 70 years, and age ≥ 70 years; BMIs were categorized into three subgroups: BMI < 25, 25 ≤ BMI < 28, and BMI ≥ 28. The difference in the relative abundance of species was compared in both sub-age and sub-BMI groups using the Kruskal‒Wallis test. Pairwise comparisons within the subgroups were performed using the Wilcoxon Mann‒Whitney U test. *p* < *0.05* was considered statistically significant.

### Co-occurrence network

Molecular ecological network analyses (MENA) were used to construct random matrix theory (RMT)-based co-occurrence networks based on the Spearman’s correlation coefficient. The network was completed using Cytoscape Version 3.9.0.

## Results

### General characteristics of microbiome distribution

Before the matching procedure, a total of 709 metagenomic samples (318 patients with CRC and 391 healthy controls from 6 projects (PRJDB4176, PRJEB10878, PRJEB27928, PRJEB7774, PRJNA397219, and PRJNA447983)) were identified based on the study criteria. After matching, we included data from 86 patients with CRC and 86 healthy controls in the subsequent data analysis. The baseline information of the matched samples is presented in Table 1, Additional file 1: Table S1, and Additional file 2: Figure S1.

**Table 1:** Baseline characteristics of patients in the GMrepo database

Variable	Before matching (n = 709)			After matching (n = 172)		
	CRC (n = 318)	Control (n = 391)	*p*	CRC (n = 86)	Control (n = 86)	*p*
Age (years, M, IQR)^†^	63 (13)	68 (12)	0.037*	64 (10)	65 (10)	0.985
Age (n, %)			0.015*			0.542
< 65 years	197 (39.1%)	307 (60.9%)		40 (47.6%)	44 (52.4%)	
≥ 65 years	101 (49.3%)	104 (50.7%)		46 (52.3%)	42 (47.7%)	
Sex (n, %)			0.003*			1.00
Male	216 (49.2%)	223 (50.8%)		56 (50%)	56 (50%)	
Female	102 (37.8%)	168 (62.2%)		30 (50%)	30 (50%)	
Country (n, %)			0.015*			1.00
Japan	36 (47.4%)	40 (47.4%)		9 (50%)	9 (50%)	
China	64 (54.2%)	54 (45.8%)		20 (50%)	20 (50%)	
United States	24 (41.2%)	33 (57.9%)		3 (50%)	3 (50%)	
Italy	22 (50%)	22 (50%)		6 (50%)	6 (50%)	
Germany	79 (37.3%)	133 (67.2%)		16 (50%)	16 (50%)	
Austria	73 (36.1%)	129 (63.9%)		32 (50%)	32 (50%)	
BMI (kg/m2, M, IQR)^†^	25.2 (5.72)	24.9 (6.14)	0.748	25.00 (6.12)	25.34 (5.86)	0.834
BMI (n, %)			0.387			0.937
Normal	148 (42.3%)	202 (57.7%)		34 (51.5%)	32 (48.5%)	
Overweight	112 (44.1%)	142 (55.9%)		28 (48.3%)	30 (51.7%)	
Obesity	38 (36.2%)	67 (63.8%)		24 (50%)	24 (50%)	

*CRC* colorectal cancer, *BMI* body weight index

* *p* < 0.05

^†^*p* value was derived from the Mann‒Whitney test in data of continuous variables with abnormal distribution (M, Median; IQR, Interquartile Range). The *p* value was derived from the chi-square test or Fisher’s exact test in data of categorical variables from colorectal cancer and healthy controls (n, %)

**Fig. 1:**
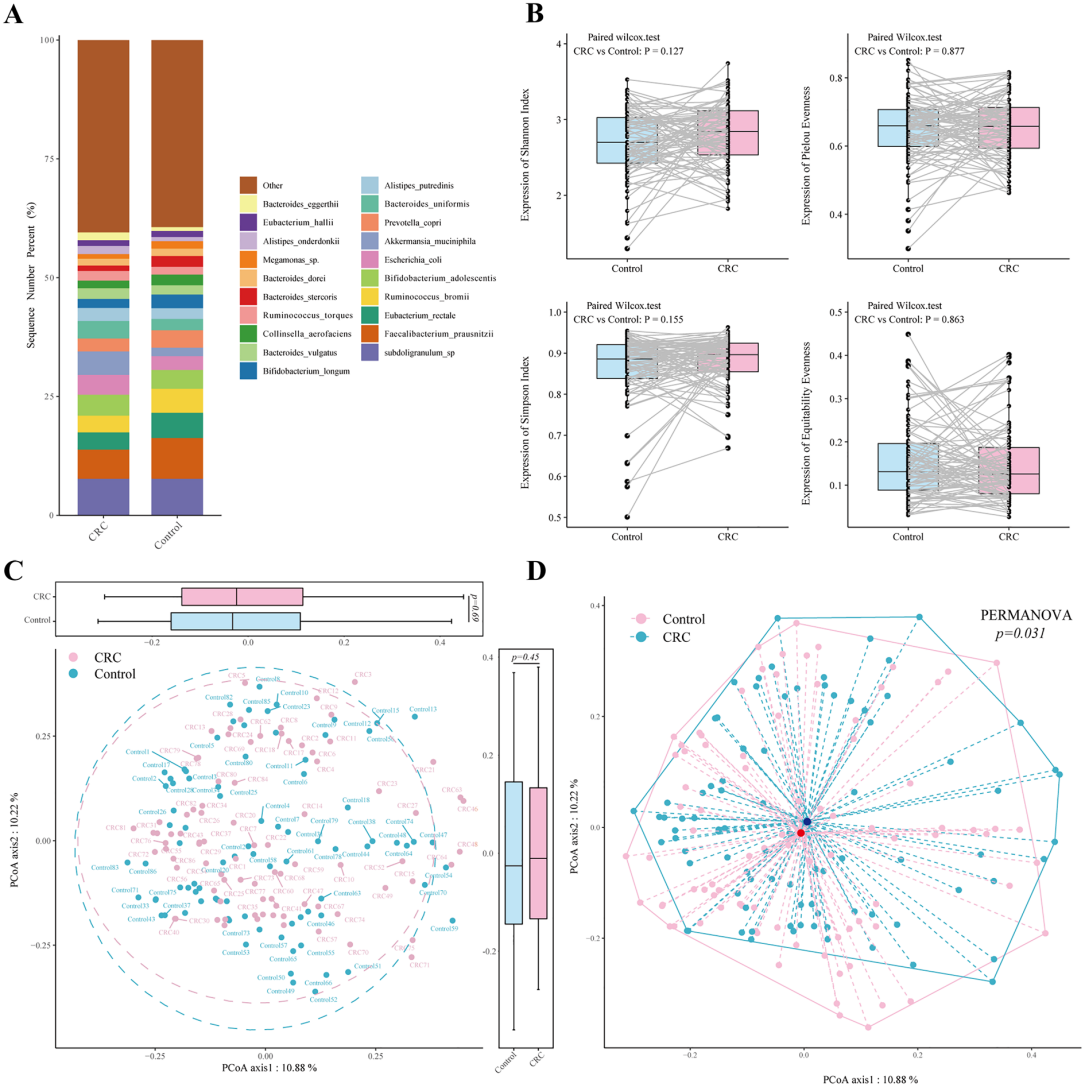
**A** The 100% stacked column chart of the relative abundance at the species level in CRC patients and healthy controls. The X-axis represents the CRC and control groups. The value of each species percentage in the Y-axis represents the mean value of relative abundance from each CRC and control cohort. The relative abundance represents the percentage of each species per sample. **B** Alpha diversity was evaluated by the Shannon index, Pielou evenness, Simpson index, and Equitability evenness. The solid lines indicate the faecal samples from CRC patients and their matched healthy controls. The difference in alpha diversity was calculated by the Wilcoxon signed‒rank test. **C** PCoAs of Bray‒Curtis distances on species composition, calculated between CRC and healthy controls. Each dot represents a patient with CRC and controls. Points clustered in light blue and pink eclipses represent the gut microbial composition of the CRC and controls, respectively. The boxplots around the PCoA plot represent the Bray‒Curtis distances of Axis1 (the top boxplot) and Axis2 (the right-sided boxplot). Differences in Bray‒Curtis distances of both Axis1 and Axis2 were calculated between the CRC and controls using the Wilcoxon Mann‒Whitney test, and p < 0.05 was considered statistically significant. **D** Visualization of dispersion differences in microbial composition between the CRC and control groups. Points in light blue and pink represent the gut microbial composition of the CRC and controls, respectively. The red and dark blue points indicate the centroids of all microbial species in each CRC and control group, respectively. The dashed line represents the spatial distance between the centroids and each sample. PERMANOVA was performed, and p < 0.05 was considered statistically significant. CRC: colorectal cancer; PCoAs: principal coordinate analyses

A total of 484 microbial species were identified. Figure 1A and Additional file 1: Table S2 show the top 20 predominant microbial species in both the CRC and control groups. Among these species, *Akkermansia muciniphila* (*A. muciniphila*) (median relative abundance 4.93% vs. 1.79%, Wilcoxon signed-rank test, *p* = *0.014)* was significantly enriched in the CRC group, whereas *Faecalibacterium prausnitzii* (*F. prausnitzii*) (6.15 vs. 8.57%, z = −2.741, *p* = *0.006*) and *Eubacterium rectale* (*E. rectale*) (3.62% vs. 5.32%, z = −1.985, p = 0.047) were more abundant in the control group. Additional file 3: Figure S2 presents the microbiome distribution of species in different subgroups according to age, region, and sex.

The alpha diversity of the gut microbiota in the CRC groups was not different from that in the control groups (Fig. 1B). For the beta diversity, there was no difference in Bray‒Curtis distances of both Axis1 *(p* = *0.690)* and Axis2 *(p* = *0.450)* in CRC versus controls during PCoA, indicating a similar species composition between the two groups (Fig. 1C). The PERMANOVA test, however, reached a significant difference (*p* = *0.031*) between the CRC and control groups (Fig. 1D), possibly due to the dispersion differences between the CRC and control groups, but such a difference is not visually significant and might not be clinically relevant.

**Fig. 2:**
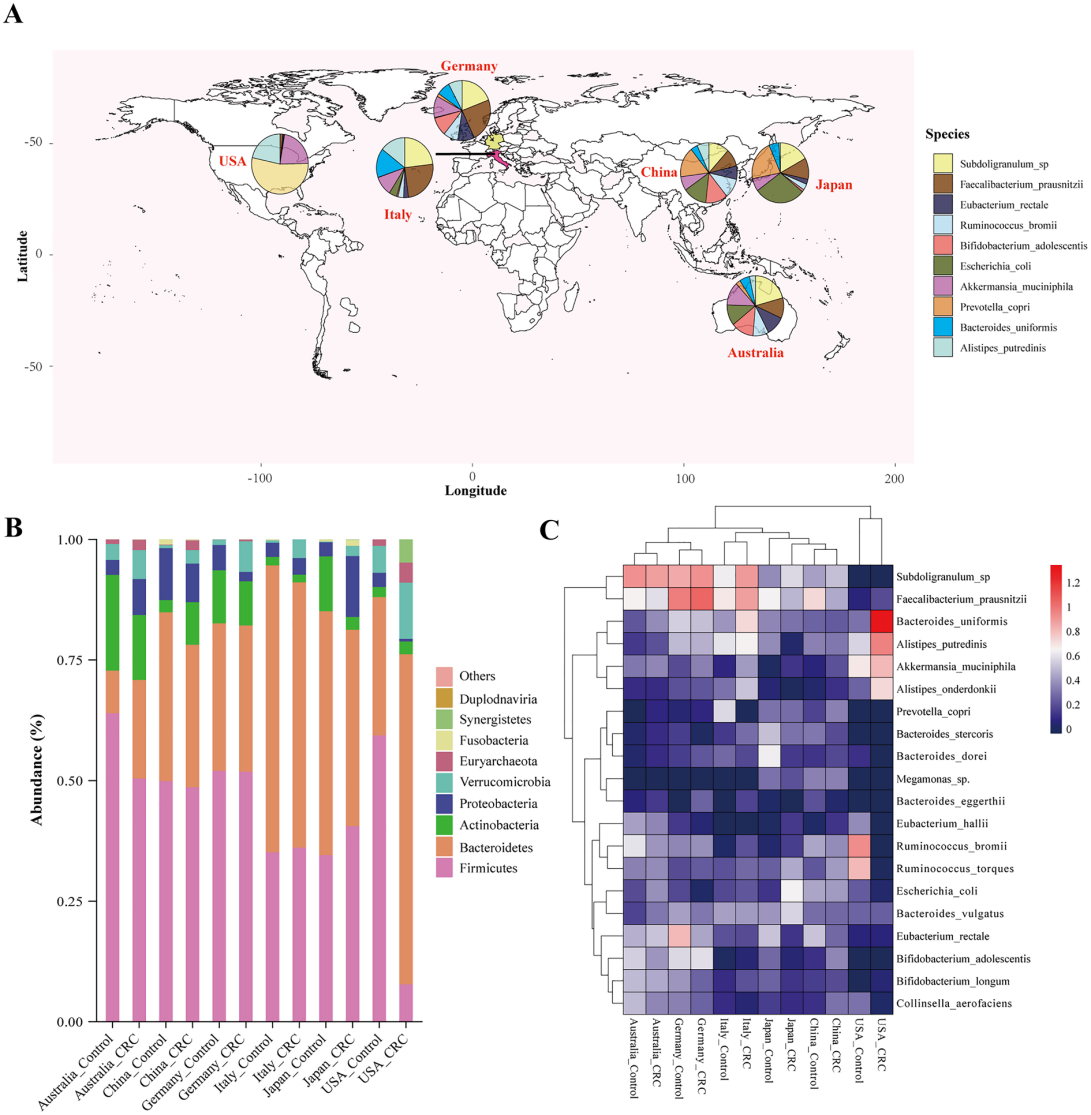
**A** Geographic distribution of samples from six different countries. Each pie chart represents the composition of the top 10 most abundant species identified in all samples distributed in CRC patients from each country. **B** The 100% stacked column chart of the relative abundance at the phylum level in CRC patients and healthy controls. The X-axis represents the subgroups of countries. The value of each phylum percentage in the Y-axis represents the mean relative abundance from each CRC and control cohort. The relative abundance represents the percentage of each species made of the organism per sample. **C** Heatmap visualization of the mean intestinal microbiota abundance in CRC patients and healthy controls based on region differences. Each column represents one subgroup based on the status of the disease in different countries. Each row represents one species ranking in the top 20 of the average relative abundance per subgroup. Values of the average relative abundance were normalized by the Z scores method. The colour scale was set based on the specific value of the average relative abundance after the Z score transformation, with red for relatively high abundance (Z scores > 0) and blue for low abundance (Z scores < 0). The greater the weights of the absolute Z score-transformed abundance values, the deeper the colour of the squares. The original values of average relative abundance per subgroup (before Z score transformation) are shown on the right of the colour scale (0, 0.2, 0.4…,1.2)

The geographic differences in the microbiome distribution is presented in Fig. 2. The distribution of the microbiome varied in different countries (Fig. 2A). At the phylum level, we noticed a consistent trend of the increased abundance of *Verrucomicrobia* and *Euryarchaeota* in the CRC groups in all six countries (Fig. 2B). In terms of certain species, *F. prausnitzii* had decreased abundance, while *A. muciniphila* had increased abundance in the CRC samples compared to that in the control samples in all subgroup regions (Fig. 2C, Additional file 1: Table S3).

**Fig. 3:**
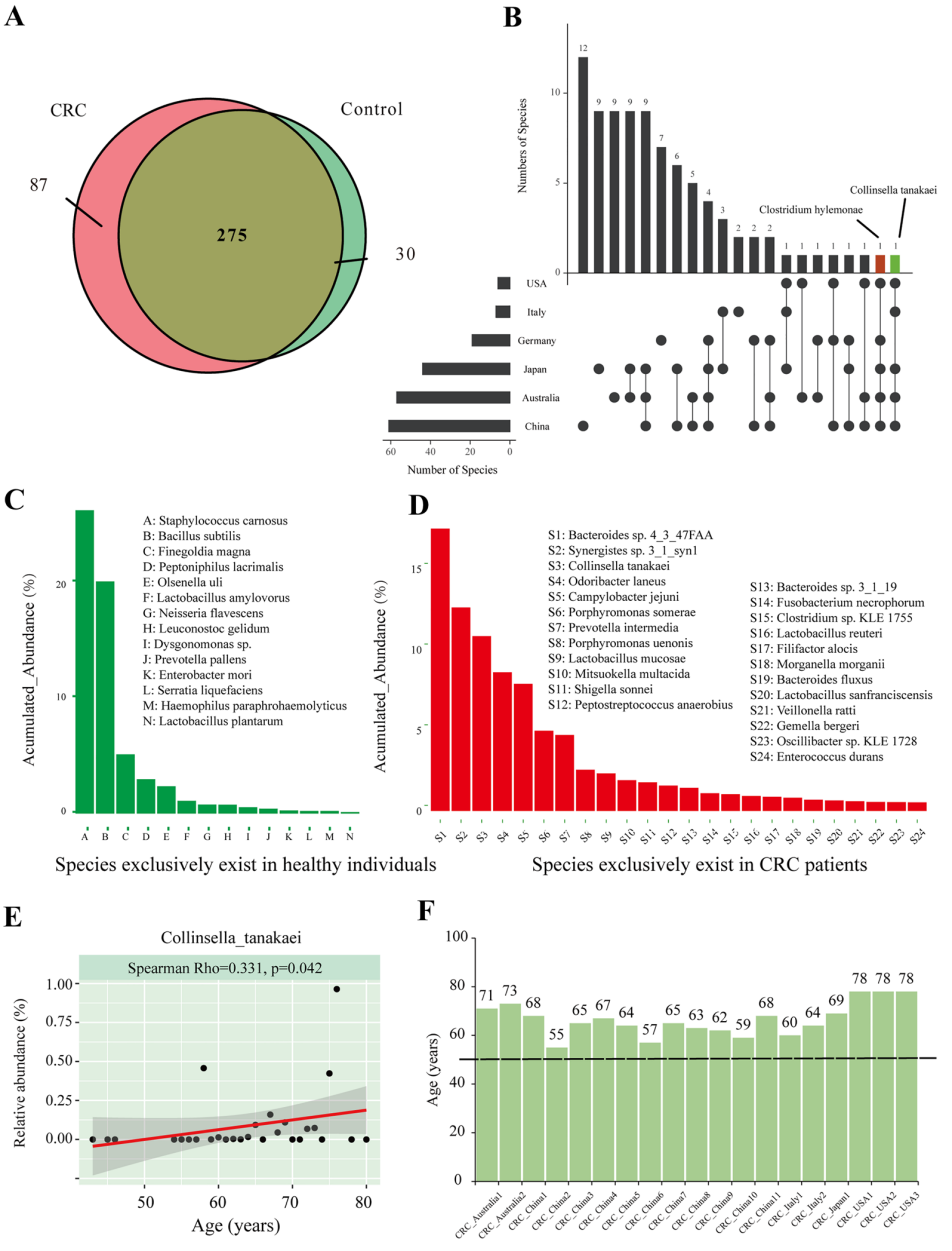
**A** Venn diagrams illustrating the number of species in CRC patients (pink) and healthy controls (light green). **B** UpSet plot of differentially distributed taxa. The left graph represents the total number of differently distributed species (X-axis) in different countries (Y-axis). The right graph represents the intersection of sets of species in multiple countries. Each column corresponds to a country or set of countries (dots connected by lines below the X-axis) containing the same species. The number of species in each set appears above the column, while countries shared are indicated in the graphic below the column. **C** Histograms of species exclusively present in healthy individuals. The X-axis represents species that are exclusively shared in healthy individuals (with an accumulated relative abundance of more than 0.5% of each sample in healthy groups). The Y-axis represents a percentage of accumulated relative abundance, which is a measure of the proportions of the microbiota composed of the organism in the healthy control group. **D** Histograms of species exclusively present in CRC individuals. The X-axis represents species that are exclusively shared in the CRC groups with an accumulated relative abundance of more than 0.5% of each sample in the CRC groups. The Y-axis represents the percentage of relative abundance in the CRC and control groups. **E** Scatter plots of *C. tanakaei* and age. The Spearman rank correlation test, p-value < 0.05 was considered statistically significant). **F** The age distributions of individuals with expression of *C. tanakaei* in the feceal samples

Within all metagenomic samples, 87 species exclusively existed in the CRC group, and 30 were in the control groups (Fig. 3A, Additional file 1: Table S4–S5). Among these 87 species, *Collinsella tanakaei* (C. *tanakaei*) and *Clostridium hylemonae* (C. *hylemonae*) were found in the CRC samples in five countries (Fig. 3B). The exclusive species were ranked by their accumulated abundance in all samples and are listed in Fig. 3C, D. To further identify the distribution characteristics of *C. tanakaei* and *C. hylemonae*, we correlated these two species with age and BMI. We found that *C. tanakaei* had a significant positive correlation with age (Spearman Rho = 0.331, *p* < *0.05*) (Fig. 3E). The distributions *of C. tanakaei* from different countries are further presented in Fig. 3F. All CRC individuals with *C. tanakaei* tended to be elderly. No significant correlations were detected between *C. tanakaei* and BMI (Spearman Rho = −0.223, *p* = *0.985), C. hylemonae* and age (Spearman Rho = 0.121, *p* = *0.475*), or *C. hylemonae* and BMI (Spearman Rho = -0.120, *p* = *0.807*).

### Identification of microbial markers for CRC diagnosis

**Fig. 4:**
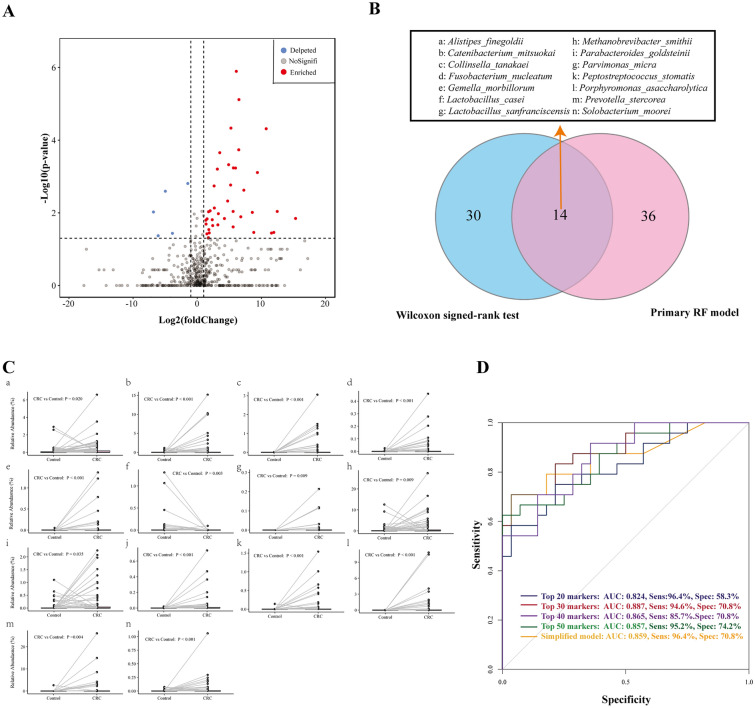
**A** Volcano plot. The log2-fold-change indicates the mean relative abundance for each species. Each dot represents one species. The grey dots represent species with no significant expression difference or species with |log2 Fold-Change|≤ 1.0 (CRC versus controls), the red dots represent depleted taxa in CRC groups compared with controls, and the light blue dots represent enriched taxa. The Wilcoxon-signed rank test was performed, and p < 0.05 was considered statistically significant. **B** Venn diagrams illustrating the number of species identified by the primary random forest model, including the top 50 microbial markers (pink) and the 44 species by the Wilcoxon signed rank test (light blue). **C** The paired scatter plot of the 14 commonly identified species between CRC and controls. **D** The area under the curve (AUC) of different models. CRC: colorectal cancer; Sens: sensitivity, Spec: specificity

Using the Wilcoxon signed-rank test, we identified 44 species with significantly different abundances between CRC patients and healthy patients with log_2_Fold-change greater than 1.0, including 5 significantly enriched species and 39 significantly depleted species in CRC versus control patients (Fig. 4A, Additional file 1: Table S6). To further determine whether the microbial species could be used as identification biomarkers for distinguishing CRC samples, we established random forest models to classify CRC patients and healthy controls. Internal cross-validation was performed. The training and validation cohort was a 7:3 split of the original data. We calculated different AUC indexes by integrating different numbers of taxa with the highest model-building importance and lowest inner subcategory bias (Additional file 4: Figures S3: A-C)**.** The top 50 microbial species are listed in Additional file 1: Table S7. The random forest models were then established by integrating different numbers of variables from the top 11 to 50 significant microbes. The AUC, sensitivity, and specificity of each model were determined and are presented in Additional file 4: Figure S3D. The median value of the AUC was 0.812 in the training cohort and 0.790 in the validation set. The model containing the top 30 microbial species showed the highest AUC (0.887) values in the training cohort, but its performance did not demonstrate superiority in the validation cohort (AUC = 0.788). Visually, the model performance tended to be more stable by integrating the top 30–50 microbial biomarkers than the top 11–29 biomarkers. Thus, we further compared the median AUC, sensitivity, and specificity of the above two model categories (Additional file 1: Tables S8-S9). The median AUC of models containing the top 30–50 species was significantly higher in the training (0.804 versus 0.784, Wilcoxon T, *p* < *0.001*) and validation (0.804 versus 0.784, Wilcoxon T, *p* < *0.001*) cohorts than that with the top 11–29 biomarkers. There was the same trend of a significantly higher median sensitivity in both the training (90.5% versus 76.9%, Wilcoxon T, p < 0.001) and validation cohorts (90.5% versus 85.7%, Wilcoxon T, *p* = *0.003*). The specificity showed no significant difference.

Next, we identified the 14 differentiated microbial species using the Wilcoxon signed rank test and the random forest algorithm. (Fig. 4B–C) Then, we established a relatively simplified model by incorporating only these 14 markers. The simplified model showed no significant differences in ROC, sensitivity, and specificity with the primary models developed from the random forest models. (Fig. 4D).

### Relationship of gut microbiota and age or BMI in CRC groups

**Fig. 5:**
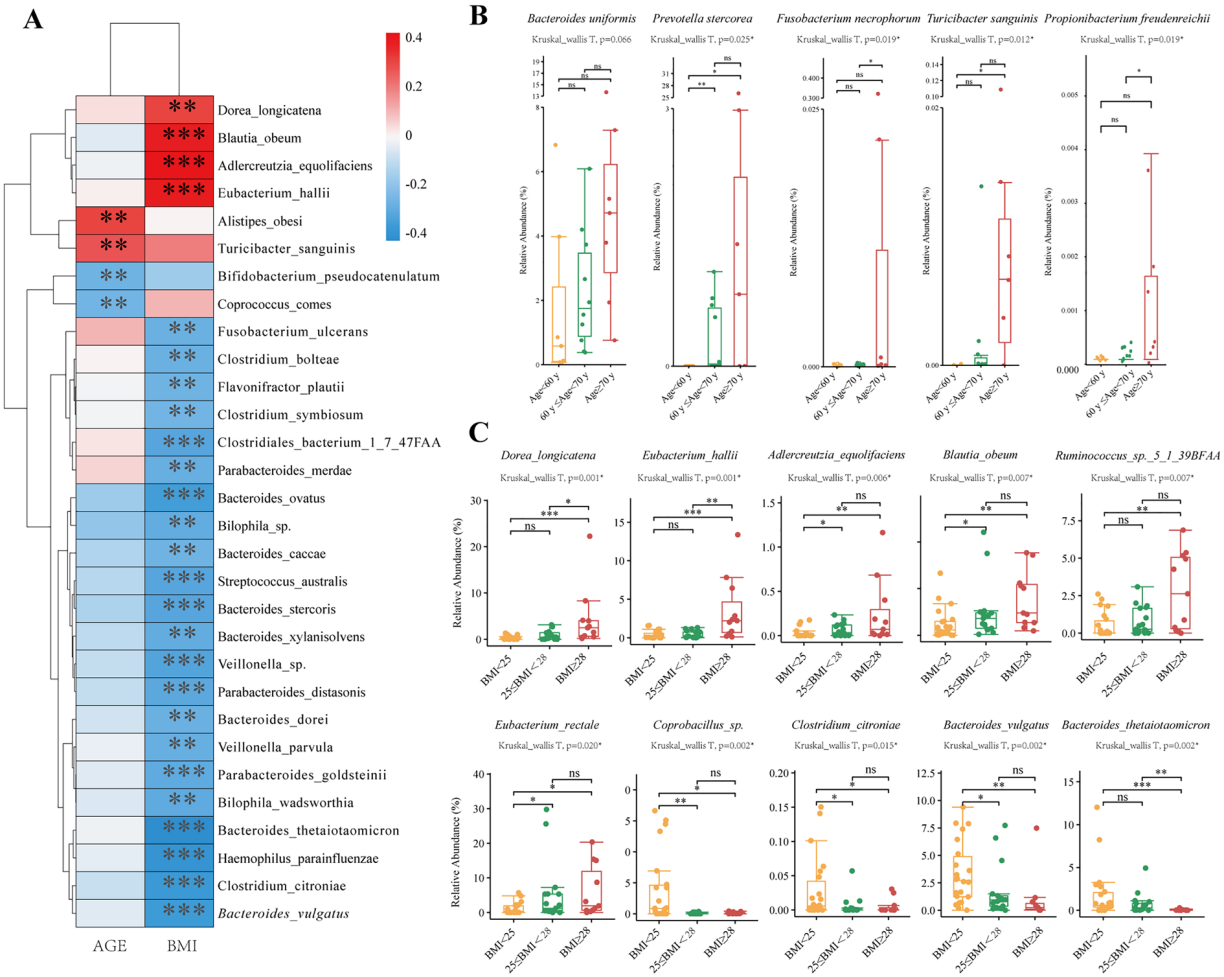
**A** Heatmap reporting correlation coefficients (Rho) and *p* values for the correlation of species and age/BMI. The row represents certain species, and the column represents age and BMI. The bar on the right side shows the colour scale reflecting the Rho values. Positive correlations of a species with age or BMI (Rho > 0) are presented as red-scale squares, whereas negative correlations (Rho < 0) are presented as blue squares. The greater the weight of the absolute Rho value is, the deeper the colour bar. "*" represents the p value of the correlation. (The Spearman rank correlation test, *, **, *** stands for p value < 0.01, 0.005 and 0.001, respectively). **B**, **C** Boxplot of species abundance distributed in different sub-age groups (**B**) and sub-BMI groups (**C**). Boxplot displays the median of relative abundances (%) with their interquartile range. The upper and lower edges of the box represent the maximum and minimum relative abundance of each microbe, respectively. Relative abundance (%) means the percentage of a microbial species composed of the organism. The difference in the relative abundance of species was compared in both sub-age and sub-BMI groups using the Kruskal‒Wallis test. Pairwise comparisons within the subgroups were calculated using the Wilcoxon Mann‒Whitney U test. A p value < 0.05 was considered statistically significant. (*, **, *** for p values < 0.01, 0.005 and 0.001, respectively)

To track the influence of age- or BMI-related differences in the gut microbiota in the CRC groups, we first applied Spearman’s correlation test. *Dorea longicatena* (*D. longicatena*) (Rho = 0.30, *p* = *0.001*), *Blautia obeum (B. obeum)* (Rho = 0.38, *p* < *0.001*), *Adlercreutzia equolifaciens* (*A. equolifaciens*) (Rho = 0.42, p < 0.001), and *Eubacterium hallii (E. hallii)* (Rho = 0.38, *p* < *0.001*) were positively correlated with BMI changes in CRC patients, whereas the other 22 strains were negatively correlated with BMI (Fig. 5A, Additional file 1: Table S10). Regarding the relationship with age, increased enrichment of *Alistipes obesi* (Rho = 0.30, *p* = *0.001*) and *Turicibacter sanguinis* (*T. sanguinis*) (Rho = 0.27, *p* = *0.005*) was significantly correlated with increased age, whereas *Bifidobacterium pseudocatenulatum* (Rho = −0.28, *p* = *0.004*) and *Coprococcus comes* Rho = −0.27, *p* = *0.005*) were negatively correlated with age in the CRC cohort.

We further tracked the principal microbial strains in the CRC group associated with age and BMI. In the sub-age groups, the distributions of 19 species were significantly different among the three groups with *p* < *0.05* by the Kruskal‒Wallis test (Additional file 5: Figure S4A). Among these species, we found a trend of increased abundance in *Prevotella stercorea* (Kruskal_wallis Test, *p·* = *0.025*), *Fusobacterium necrophorum* (*p* = *0.025*), *T. sanguinis* (*p* = *0.012*), and *Propionibacterium freudenreichii* (*p* = *0.019*), which were associated with stepped growth of age in the subgroups (Fig. 5B). *Bacteroides uniformis (B. uniformis)* had an increased abundance in the three sub-age groups but did not reach statistical significance. To further identify microbes significantly associated with age, we utilized a random forest algorithm (Additional file 1: Figure S4B). *B. uniformis* was identified as one of the top 12 candidate microbes significantly associated with age (with a value of 18.0 for the increase in node purity).

Considering that the number of patients with obesity (BMI exceeding 30) was only two in the current CRC cohort, we slightly adjusted the cut-off value in BMI subgroups for better statistical comparisons. With stepped growth of BMI in the three subgroups, there was a trend of an increased abundance of *D. longicatena* (Kruskal‒Wallis test, *p* = *0.001*), *E. hallii* (*p* = *0.001*), *A. equolifaciens* (p = 0.006), *B. obeum* (*p* = *0.007*), and *Ruminococcus sp.* (*p* = *0.007*), while there was a trend of decreased abundance of *Coprobacillus sp.* (*p* = *0.002*), *Clostridium citroniae* (*p* = *0.015*), *Bacteroides vulgatus* (*p* = *0.015*), and *Bacteroides thetaiotaomicron* (*p* = *0.002*) (Fig. 5C, Additional file 6: Figure S5A). The top 12 important BMI-related species are presented in Fig. 4C, in which the abundance of *Ruminococcus sp*, *D. longicatena*, *Anaerostipes hadrus*, *A. equolifaciens*, and *E. rectale* tended to increase after BMI exceeded 30 kg/m^2^. (Additional file 6: Figure S5B).

### Establishment of the microbial interaction network

**Fig. 6:**
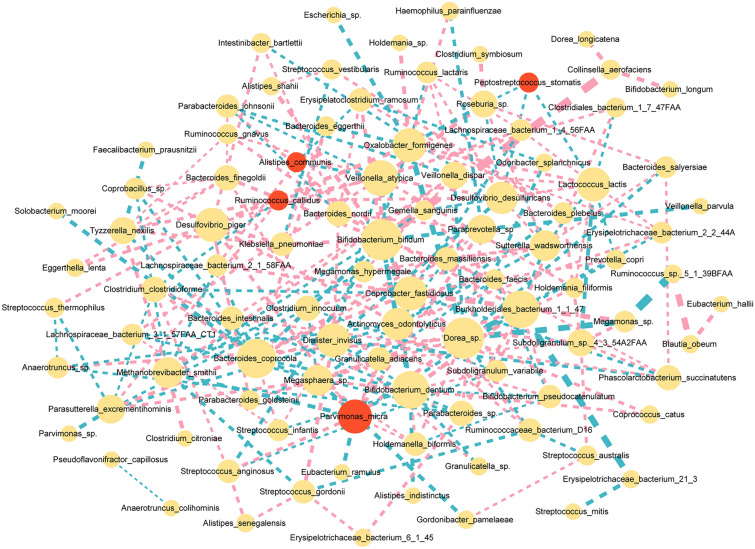
Co-occurrence network visualization of the interactions among different species. The lines connecting nodes (edges) represent a positive (pink) or negative (light blue) co-occurrence relationship. The width of the edges represents the strength of the correlation, and the size of the circle represents the degree of interaction with other species. The red colour represents significant CRC-enriched species. The yellow colour represents species with no significant association with CRC status

Network analysis was performed based on the relative abundance of 484 taxa in both groups to explore the co-occurrence relationship among these microbial species (Fig. 6). The network was composed of 97 nodes with a total of 235 links. Regarding CRC-enriched specifics, *Parvimonas micra* (*P. micra*) showed the most frequent interaction with other species: *Holdemanella biformis* (positive relation, clustering coefficient (cc) 0.40), *Desulfovibrio piger*, (positive relation, cc 0.09), *Burkholderiales bacterium* (negative relation, cc 0.06), and *Methanobrevibacter smithii* (negative relation, cc 0.03). *Peptostreptococcus stomatis* was negatively related to *Desulfovibrio desulfuricans* (cc 0.67), which was also positively related to another CRC-enriched species, *Ruminococcus callidus* (cc 0.33).

## Discussion

To assess differences in the gut microbe composition in CRC individuals versus healthy controls, we designed a paired-sample study based on the metagenomic data from the GMrepo database. To our knowledge, this is the first bioinformatics analysis using a public database instead of the recruitment and enrolment of CRC subjects. This type of study (1) enables the comparison of gut microbiota among CRC patients from different countries and (2) is beneficial for identifying new CRC-enriched microorganisms commonly present worldwide, regardless of regional differences.

Using the metagenomic data in our study, we precisely identified microorganisms that were enriched or depleted in CRC patients at the species level. First, we confirmed the novel associations of CRC with several enriched species as previously reported, including *F. nucleatum* and species belonging to the genera *Parvimonas, Peptostreptococcus, Porphyromonas,* and *Prevotella*. [4, 21, 22] Within the species that were enriched in CRC patients, we noticed that *P. micra* had the highest mean decrease accuracy in the random forest model, and it also had frequent interactions with other species in the co-occurrence network. This finding was consistent with previous studies that reported that *P. micra* plays a key role in CRC formation [3, 25]. Another species we noticed is *A. muciniphila.* We found that *A. muciniphila* was overrepresented in CRC patients compared with healthy controls. According to our subgroup results, the CRC enrichment tendency of *A. muciniphila* was present in all six countries. This result is consistent with several previous studies [24–26] in which *A. muciniphila* was considered a CRC-enriched biomarker that could promote CRC formation by triggering inflammation and intestinal epithelial cell proliferation. However, when we chose biomarkers in the random forest model, *A. muciniphila* was not a statistically significant candidate predictor. Some studies have reported anti-tumorigenesis features of *A. muciniphila* [27], which would suggest a contradictory role of *A. muciniphila* in CRC formation. We also detected a slight decreasing trend of *A. muciniphila* in CRC patients with obesity. Therefore, we assume that the abundance of *A. muciniphila* might fluctuate due to its potential interaction with other environmental factors. It cannot be considered a single biomarker to confirm CRC diagnosis.

Although the distributions of gut microbes differ among different countries, we identified some new species exclusively existing in CRC patients which are commonly present in patients from most countries. *C. tanakaei* is a novel taxon that was exclusively found in CRC samples from five different countries, and its accumulated abundance ranks third among all CRC-specific species. Although *C. tanakaei* has not been reported to have direct associations with CRC, it has a previously reported association with a gene called 12-beta-HSDH, which is related to CRC progression [28]. In addition, this species has been shown to interact with related metabolites, such as lactate, acetate, and formate. These metabolites are major end products of glucose fermentation, which might be involved in primary and metastatic colon cancer cells [29]. Interestingly, we also noticed that this species had increased relative abundance with increasing age and was more likely to be detected in older CRC patients. Because there are currently no reports of the direct involvement of *C. tanakaei* in CRC pathogenesis, further mechanistic studies are needed to validate its causal relationship with CRC, especially in old CRC patients.

In addition, we also investigated the interaction between the gut microbiota and the following two important CRC-associated factors: age and BMI. We found that *B. uniformis* is not only a significant CRC-enriched species, but also an age-discriminatory bacterial taxon. In our study, *B. uniformis* showed an increase in abundance with increasing age, and this trend was more apparent in patients over 70 years old. There is still no promising evidence on the role of *B. uniformis* in CRC activity. Wang [30] found an increased abundance of *B. uniformis* in healthy volunteers. Justesen [31], however, reported an enriched abundance and biomarker potential of *B. uniformis* in CRC diagnosis. No research has investigated the role of *B. uniformis* in senescence progression. Thus, future studies should focus on investigating the association between this species and age-related diseases. In addition, we noticed that *D. longicatena*, *A. equolifaciens,* and *E. hallii* had positive associations with BMI, which was validated by all statistical methods. *D. longicatena* and *E. hallii* are obesity-related microorganisms reported by previous studies [32, 33]. Interestingly, we detected that *E. hallii* also had an indirect positive relationship with *Ruminococcus sp.* in our cooccurrence network analysis. *Ruminococcus sp.* was another species identified by both the random forest method and subgroup difference analysis to be positively related to BMI levels, especially in obese CRC patients. Further studies should focus on the possible interaction among these obese-clustered bacteria in CRC patients.

To further develop a diagnostic panel for CRC screening, we established 40 models by integrating different numbers of significant predictors from the top 11 to 50 ranked microbes identified by the random forest algorithm. Visually, based on the curves, the model performance tends to be more stable in models with the top 30–50 microbial biomarkers. The median AUC and sensitivity were significantly higher in models from the top 30–50 microbes than in those from the top 11–29 markers in both the training and validation cohorts. Therefore, using the models from the top 30–50 microbes may have a better and more stable model performance for CRC diagnosis. To simplify the model, we established another model using 14 commonly identified species by two different statistical algorithms. The simplified model showed comparable performance with the primary random forest models; however, as the sample size for developing the model is relatively small, these models still need prospective validations to confirm their clinical application prospects.

Although the abundance of some species was significantly enriched or depleted in CRC patients compared with controls, these species were not all selected as significant predictors in the final prediction model. This indicates that a more complex microbial interaction possibly triggers the formation of CRC rather than just a single species. Thus, a microbial panel consisting of a collection of species with significant contributions should be applied clinically as a non-invasive biomarker, rather than using a single microorganism.

Our study has some limitations. First, it is a case‒control study with a relatively small sample size. Although we primarily performed quantitative comparisons based on a paired-sample design, it would be better to reduce the individualized differences in other environmental factors by recruiting prospective cohorts, enabling tracking of the microbial changes in the same person before and after CRC. Second, we were unable to access the detailed data of clinical characteristics such as the tumour size, location, stage, and the results of other diagnostic tests due to the unavailability of this information in the GMrepo database. Third, we applied only paired sample matching as an approach to eliminate batch effects from biological origins, and batch effects originating from technical and computational sources may still exist. Additionally, the prediction models we established, integrating only microbial biomarkers for CRC screening, may be less accurate in real-world clinical practice. Thus, it would be better to add more detailed clinical variables associated with CRC in the future development of the microbiota database, which could promote further analysis of the interaction between the gut microbiota and other environmental factors. It may also be helpful to establish more comprehensive prediction models for CRC screening based on variables that are clinically relevant in CRC pathogenesis.

In conclusion, we demonstrated gut microbial changes in CRC patients and established a microbial panel as a non-invasive method for CRC diagnosis. The identification of key species and their associated genes should be further investigated to determine the relative causality of microbial organisms and CRC development. This study may inspire more mechanistic studies to interrogate the causal molecules in microbiome-linked CRC carcinogenesis.

## Supplementary Information

**Additional file 1: Table S1.** Clinical Information of the paired samples in the CRC and healthy controls. **Table S2.** The top 20 abundant species in the all paired-samples. **Table S3.** The top 20 abundant species in the CRC and controls distributed in six countries. **Table S4.** Species exclusively exists in the CRC groups. **Table S5.** Species exclusively exists in the Control groups. **Table S6.** Species significantly different in abundance in the CRC and healthy patients (with LogFC＞1). **Table S7.** The top 50 significant species identified in the Random Forest Model. **Table S8.**The performance of prediction models by integrating different numbers of microbe species from the top 11 to 50 microbes. **Table S9.** Performance of prediction models by integrating different numbers of microbe species from the top 11 to 50 microbes. **Table S10.** Correlation coefficients (Rho) and p values for the Spearmen correlation of different species and age/BMI.

**Additional file 2: Figure S1.** The flow chart of the study design.

**Additional file 3: Figure S2.** The 100% stacked column chart of relative abundance of the top 20 dominant species in CRC patients and healthy controls based on sub-BMI (A), sex (B), and region (C). The X axis represents different subgroups. The value of each species percentage in the Y-axis represents the mean of relative abundance from each subgroup. The relative abundance represents the percentage of each species made of the organism per sample.

**Additional file 4: Figure S3.** A: Random forest model of the 30 representative microbial biomarkers to predict CRC based on their mean decrease scores of the optimal model performance. The red square on the right side of each species represents the enrichment of this species in CRC groups, whereas the green square represents the enrichment of this species in controls. B-C: The area under the curve (AUC) of different models. The training and validation cohort is a 7:3 split of original data. Different AUC indexes by integrating different numbers of taxa with the highest model-building importance and lowest inner subcategory bias. D: The performance of prediction models by integrating different numbers of microbe species from the top 11 to 50 microbes. The X-axis represents the number of variables in each prediction model from the top 11 to the top 50 microbes. The Y-axis represents the value of AUC (the green curves), sensitivity (the yellow curves), and specificity (the blue curves) of each cohort.

**Additional file 5: Figure S4.** A: Species with significantly difference (Kruskal wallis Test, p<0.05) in distributions among the three sub-age groups. The boxplot displays the median of relative abundances (%) with their interquartile range. The upper and lower edge of the box represents the maximum and minimum relative abundance in each microbe, respectively. Relative abundance (%) means the percentage of a microbial species composed of the organism. The pair-wise comparisons within the subgroups were calculated using the Wilcoxon Mann-Whitney. p-value＜0.05 was considered statistical significance. (*, **, *** for p-values < 0.01, 0.005 and 0.001, respectively). B: The top 12 important species significantly associated with age in CRC patients (identified by the random forest algorithm). The Y-axis represents the relative abundance (Log2 transformed) of each species. The X-axis represents age as continuous variable. The relative abundance represents the percentage of each species made of the organism per sample. The Increased Node Impurity Index (IncNodePurity) was listed on the top of each species. Microbes with higher values of the IncNodePurity are considered to have higher association with age.

**Additional file 6: Figure S5.** A: Species with significantly difference (Kruskal wallis Test, p<0.05) in distributions among the three sub-BMI groups. The boxplot displays the median of relative abundances (%) with their interquartile range. The upper and lower edge of the box represents the maximum and minimum relative abundance in each microbe, respectively. Relative abundance (%) means the percentage of a microbial species composed of the organism. The pair-wise comparisons within the subgroups were calculated using the Wilcoxon Mann-Whitney. p-value＜0.05 was considered statistical significance. (*, **, *** for p-values < 0.01, 0.005 and 0.001, respectively). B: The top 12 important species significantly associated with age in CRC patients (identified by the random forest algorithm). The Y-axis represents the relative abundance (Log2 transformed) of each species. The X-axis represents age as continuous variable. The relative abundance represents the percentage of each species made of the organism per sample. The Increased Node Impurity Index (IncNodePurity) was listed on the top of each species. Microbes with higher values of the IncNodePurity are considered to have higher association with BMI.

## Data Availability

All data and materials during the current study are available from the corresponding author on reasonable request.
