# Supplementary material for: Metagenomic analysis of the interaction between the gut microbiota and colorectal cancer: a paired-sample study based on the GMrepo database
Source: Gut Pathog. 2022 Dec 23;14:48. doi: 10.1186/s13099-022-00527-8 (PMC9784093; doi:10.1186/s13099-022-00527-8)
Supplement: Supplementary file 1 — Additional file 1: Table S1. Clinical Information of the paired samples in the CRC and healthy controls. Table S2. The top 20 abundant species in the all paired-samples. Table S3. The top 20 abundant species in the CRC and controls distributed in six countries. Table S4. Species exclusively exists in the CRC groups. Table S5. Species exclusively exists in the Control groups. Table S6. Species significantly different in abundance in the CRC and healthy patients (with LogFC＞1). Table S7. The top 50 significant species identified in the Random Forest Model. Table S8.The performance of prediction models by integrating different numbers of microbe species from the top 11 to 50 microbes. Table S9. Performance of prediction models by integrating different numbers of microbe species from the top 11 to 50 microbes. Table S10. Correlation coefficients (Rho) and p values for the Spearmen correlation of different species and age/BMI. [file 13099_2022_527_MOESM1_ESM.docx]

**Additional fileTable List**

Table S1. Clinical Information of the paired samples in the CRC and healthy controls.

Table S2. The top 20 abundant species in the all paired-samples.

Table S3. The top 20 abundant species in the CRC and controls distributed in six countries.

Table S4. Species exclusively exists in the CRC groups.

Table S5. Species exclusively exists in the Control groups.

Table S6. Species significantly different in abundance in the CRC and healthy patients (with LogFC＞1).

Table S7. The top 50 significant species identified in the Random Forest Model.

Table S8.The performance of prediction models by integrating different numbers of microbe species from the top 11 to 50 microbes.

Table S9. Performance of prediction models by integrating different numbers of microbe species from the top 11 to 50 microbes.

Table S10. Correlation coefficients (Rho) and p values for the Spearmen correlation of different species and age/BMI.

**Suppl. Figure Legends**

Figure S1. The flow chart of the study design.

Figure S2. The 100% stacked column chart of relative abundance of the top 20 dominant species in CRC patients and healthy controls based on sub-BMI (A), sex (B), and region (C). The X axis represents different subgroups. The value of each species percentage in the Y-axis represents the mean of relative abundance from each subgroup. The relative abundance represents the percentage of each species made of the organism per sample.

Figure S3**.** A: Random forest model of the 30 representative microbial biomarkers to predict CRC based on their mean decrease scores of the optimal model performance. The red square on the right side of each species represents the enrichment of this species in CRC groups, whereas the green square represents the enrichment of this species in controls. B-C: The area under the curve (AUC) of different models. The training and validation cohort is a 7:3 split of original data. Different AUC indexes by integrating different numbers of taxa with the highest model-building importance and lowest inner subcategory bias. D: The performance of prediction models by integrating different numbers of microbe species from the top 11 to 50 microbes. The X-axis represents the number of variables in each prediction model from the top 11 to the top 50 microbes. The Y-axis represents the value of AUC (the green curves), sensitivity (the yellow curves), and specificity (the blue curves) of each cohort.

Figure S4. A: Species with significantly difference (Kruskal wallis Test, p<0.05) in distributions among the three sub-age groups. The boxplot displays the median of relative abundances (%) with their interquartile range. The upper and lower edge of the box represents the maximum and minimum relative abundance in each microbe, respectively. Relative abundance (%) means the percentage of a microbial species composed of the organism. The pair-wise comparisons within the subgroups were calculated using the Wilcoxon Mann-Whitney. p-value＜0.05 was considered statistical significance. (*, **, *** for p-values < 0.01, 0.005 and 0.001, respectively). B: The top 12 important species significantly associated with age in CRC patients (identified by the random forest algorithm). The Y-axis represents the relative abundance (Log2 transformed) of each species. The X-axis represents age as continuous variable. The relative abundance represents the percentage of each species made of the organism per sample. The Increased Node Impurity Index (IncNodePurity) was listed on the top of each species. Microbes with higher values of the IncNodePurity are considered to have higher association with age.

Figure S5. A: Species with significantly difference (Kruskal wallis Test, p<0.05) in distributions among the three sub-BMI groups. The boxplot displays the median of relative abundances (%) with their interquartile range. The upper and lower edge of the box represents the maximum and minimum relative abundance in each microbe, respectively. Relative abundance (%) means the percentage of a microbial species composed of the organism. The pair-wise comparisons within the subgroups were calculated using the Wilcoxon Mann-Whitney. p-value＜0.05 was considered statistical significance. (*, **, *** for p-values < 0.01, 0.005 and 0.001, respectively). B: The top 12 important species significantly associated with age in CRC patients (identified by the random forest algorithm). The Y-axis represents the relative abundance (Log2 transformed) of each species. The X-axis represents age as continuous variable. The relative abundance represents the percentage of each species made of the organism per sample. The Increased Node Impurity Index (IncNodePurity) was listed on the top of each species. Microbes with higher values of the IncNodePurity are considered to have higher association with BMI.

**Table S1 Clinical Information of the paired samples in the CRC and healthy controls.**

| **Project ID** | **Run ID** | **Disease** | **Country** | **Sex** | **Age** | **BMI status** | **BMI** | **Matched ID** |
| --- | --- | --- | --- | --- | --- | --- | --- | --- |
| PRJDB4176 | DRR127774 | CRC | Japan | Female | 69 | Overweight | 25.6369 | 1 |
| PRJDB4176 | DRR127546 | Control | Japan | Female | 67 | Overweight | 26.0417 | 1 |
| PRJDB4176 | DRR127647 | CRC | Japan | Female | 67 | Normal | 20.1746 | 2 |
| PRJDB4176 | DRR127583 | Control | Japan | Female | 65 | Normal | 19.8189 | 2 |
| PRJDB4176 | DRR127718 | CRC | Japan | Female | 75 | Normal | 22.2222 | 3 |
| PRJDB4176 | DRR127613 | Control | Japan | Female | 75 | Normal | 22.6667 | 3 |
| PRJDB4176 | DRR127514 | CRC | Japan | Male | 78 | Normal | 22.68 | 4 |
| PRJDB4176 | DRR127616 | Control | Japan | Male | 78 | Normal | 22.8624 | 4 |
| PRJDB4176 | DRR127666 | CRC | Japan | Male | 78 | Overweight | 25.9095 | 5 |
| PRJDB4176 | DRR127692 | Control | Japan | Male | 76 | Overweight | 25.9695 | 5 |
| PRJDB4176 | DRR127476 | CRC | Japan | Male | 64 | Normal | 22.46 | 6 |
| PRJDB4176 | DRR127707 | Control | Japan | Male | 63 | Normal | 22.0581 | 6 |
| PRJDB4176 | DRR127720 | CRC | Japan | Female | 73 | Normal | 20.5457 | 7 |
| PRJDB4176 | DRR127776 | Control | Japan | Female | 73 | Normal | 20.7031 | 7 |
| PRJDB4176 | DRR127507 | CRC | Japan | Male | 65 | Normal | 21.3039 | 8 |
| PRJDB4176 | DRR162775 | Control | Japan | Male | 65 | Normal | 21.5645 | 8 |
| PRJDB4176 | DRR127478 | CRC | Japan | Female | 66 | Normal | 22.6003 | 9 |
| PRJDB4176 | DRR162776 | Control | Japan | Female | 66 | Normal | 22.3478 | 9 |
| PRJEB10878 | ERR1018212 | CRC | China | Female | 61 | Normal | 22 | 10 |
| PRJEB10878 | ERR1018203 | Control | China | Female | 58 | Normal | 21.6 | 10 |
| PRJEB10878 | ERR1018304 | CRC | China | Male | 68 | Normal | 21.5 | 11 |
| PRJEB10878 | ERR1018205 | Control | China | Male | 68 | Normal | 21.7 | 11 |
| PRJEB10878 | ERR1018199 | CRC | China | Male | 55 | Normal | 22.2 | 12 |
| PRJEB10878 | ERR1018233 | Control | China | Male | 58 | Normal | 22.3 | 12 |
| PRJEB10878 | ERR1018196 | CRC | China | Male | 65 | Overweight | 24.8 | 13 |
| PRJEB10878 | ERR1018234 | Control | China | Male | 64 | Overweight | 24.8 | 13 |
| PRJEB10878 | ERR1018185 | CRC | China | Male | 64 | Normal | 23.1 | 14 |
| PRJEB10878 | ERR1018236 | Control | China | Male | 62 | Normal | 23.5 | 14 |
| PRJEB10878 | ERR1018187 | CRC | China | Female | 67 | Overweight | 24.7 | 15 |
| PRJEB10878 | ERR1018237 | Control | China | Female | 69 | Overweight | 24.9 | 15 |
| PRJEB10878 | ERR1018220 | CRC | China | Male | 56 | Overweight | 25.9 | 16 |
| PRJEB10878 | ERR1018238 | Control | China | Male | 55 | Overweight | 26.4 | 16 |
| PRJEB10878 | ERR1018190 | CRC | China | Male | 61 | Overweight | 25.2 | 17 |
| PRJEB10878 | ERR1018240 | Control | China | Male | 59 | Overweight | 25.6 | 17 |
| PRJEB10878 | ERR1018194 | CRC | China | Female | 72 | Overweight | 26.3 | 18 |
| PRJEB10878 | ERR1018242 | Control | China | Female | 71 | Overweight | 26.7 | 18 |
| PRJEB10878 | ERR1018228 | CRC | China | Male | 64 | Overweight | 24.8 | 19 |
| PRJEB10878 | ERR1018245 | Control | China | Male | 64 | Overweight | 24.7 | 19 |
| PRJEB10878 | ERR1018223 | CRC | China | Male | 57 | Normal | 23.2 | 20 |
| PRJEB10878 | ERR1018250 | Control | China | Male | 58 | Normal | 23.1 | 20 |
| PRJEB10878 | ERR1018312 | CRC | China | Male | 63 | Normal | 22.5 | 21 |
| PRJEB10878 | ERR1018252 | Control | China | Male | 65 | Normal | 22.4 | 21 |
| PRJEB10878 | ERR1018189 | CRC | China | Female | 59 | Normal | 20.7 | 22 |
| PRJEB10878 | ERR1018259 | Control | China | Female | 62 | Normal | 21.1 | 22 |
| PRJEB10878 | ERR1018213 | CRC | China | Male | 63 | Overweight | 24.4 | 23 |
| PRJEB10878 | ERR1018260 | Control | China | Male | 64 | Overweight | 24.1 | 23 |
| PRJEB10878 | ERR1018225 | CRC | China | Male | 62 | Normal | 22.7 | 24 |
| PRJEB10878 | ERR1018265 | Control | China | Male | 65 | Normal | 22.5 | 24 |
| PRJEB10878 | ERR1018311 | CRC | China | Male | 55 | Overweight | 25.8 | 25 |
| PRJEB10878 | ERR1018270 | Control | China | Male | 58 | Overweight | 26.3 | 25 |
| PRJEB10878 | ERR1018310 | CRC | China | Male | 68 | Normal | 23.7 | 26 |
| PRJEB10878 | ERR1018272 | Control | China | Male | 69 | Normal | 23.8 | 26 |
| PRJEB10878 | ERR1018191 | CRC | China | Male | 60 | Normal | 23.2 | 27 |
| PRJEB10878 | ERR1018274 | Control | China | Male | 59 | Normal | 23.7 | 27 |
| PRJEB10878 | ERR1018289 | CRC | China | Male | 62 | Normal | 23.5 | 28 |
| PRJEB10878 | ERR1018276 | Control | China | Male | 61 | Normal | 23.7 | 28 |
| PRJEB10878 | ERR1018294 | CRC | China | Male | 59 | Overweight | 24.5 | 29 |
| PRJEB10878 | ERR1018278 | Control | China | Male | 58 | Overweight | 24.5 | 29 |
| PRJEB27928 | ERR2726482 | CRC | Germany | Female | 63 | Obesity | 28.8 | 30 |
| PRJEB27928 | ERR2726405 | Control | Germany | Female | 62 | Obesity | 28.5 | 30 |
| PRJEB27928 | ERR2726633 | CRC | Germany | Female | 60 | Normal | 21.9 | 31 |
| PRJEB27928 | ERR2726421 | Control | Germany | Female | 59 | Normal | 22.1 | 31 |
| PRJEB27928 | ERR2726503 | CRC | Germany | Female | 60 | Normal | 21.9 | 32 |
| PRJEB27928 | ERR2726434 | Control | Germany | Female | 57 | Normal | 22.2 | 32 |
| PRJEB27928 | ERR2726540 | CRC | Germany | Female | 63 | Overweight | 25.8 | 33 |
| PRJEB27928 | ERR2726436 | Control | Germany | Female | 60 | Overweight | 25.8 | 33 |
| PRJEB27928 | ERR2726410 | CRC | Germany | Female | 63 | Overweight | 25.8 | 34 |
| PRJEB27928 | ERR2726446 | Control | Germany | Female | 61 | Overweight | 25.4 | 34 |
| PRJEB27928 | ERR2726657 | CRC | Germany | Male | 59 | Overweight | 26.5 | 35 |
| PRJEB27928 | ERR2726471 | Control | Germany | Male | 61 | Overweight | 27 | 35 |
| PRJEB27928 | ERR2726437 | CRC | Germany | Male | 61 | Overweight | 25.2 | 36 |
| PRJEB27928 | ERR2726475 | Control | Germany | Male | 64 | Overweight | 25.4 | 36 |
| PRJEB27928 | ERR2726501 | CRC | Germany | Female | 62 | Normal | 21.3 | 37 |
| PRJEB27928 | ERR2726485 | Control | Germany | Female | 64 | Normal | 21.1 | 37 |
| PRJEB27928 | ERR2726476 | CRC | Germany | Female | 61 | Overweight | 24.1 | 38 |
| PRJEB27928 | ERR2726493 | Control | Germany | Female | 58 | Normal | 23.8 | 38 |
| PRJEB27928 | ERR2726655 | CRC | Germany | Male | 73 | Overweight | 26.6 | 39 |
| PRJEB27928 | ERR2726533 | Control | Germany | Male | 70 | Overweight | 26.7 | 39 |
| PRJEB27928 | ERR2726480 | CRC | Germany | Female | 63 | Obesity | 28.8 | 40 |
| PRJEB27928 | ERR2726535 | Control | Germany | Female | 62 | Obesity | 28.5 | 40 |
| PRJEB27928 | ERR2726530 | CRC | Germany | Male | 59 | Overweight | 26.5 | 41 |
| PRJEB27928 | ERR2726601 | Control | Germany | Male | 61 | Overweight | 27 | 41 |
| PRJEB27928 | ERR2726567 | CRC | Germany | Male | 61 | Overweight | 25.2 | 42 |
| PRJEB27928 | ERR2726605 | Control | Germany | Male | 64 | Overweight | 25.4 | 42 |
| PRJEB27928 | ERR2726631 | CRC | Germany | Female | 62 | Normal | 21.3 | 43 |
| PRJEB27928 | ERR2726615 | Control | Germany | Female | 64 | Normal | 21.1 | 43 |
| PRJEB27928 | ERR2726606 | CRC | Germany | Female | 61 | Overweight | 24.1 | 44 |
| PRJEB27928 | ERR2726623 | Control | Germany | Female | 58 | Normal | 23.8 | 44 |
| PRJEB27928 | ERR2726524 | CRC | Germany | Male | 73 | Overweight | 26.6 | 45 |
| PRJEB27928 | ERR2726663 | Control | Germany | Male | 70 | Overweight | 26.7 | 45 |
| PRJEB7774 | ERR688548 | CRC | Austria | Male | 68 | Obesity | 32.25 | 46 |
| PRJEB7774 | ERR688359 | Control | Austria | Male | 68 | Obesity | 32 | 46 |
| PRJEB7774 | ERR688641 | CRC | Austria | Male | 69 | Obesity | 29.94 | 47 |
| PRJEB7774 | ERR688363 | Control | Austria | Male | 66 | Obesity | 30.3 | 47 |
| PRJEB7774 | ERR688433 | CRC | Austria | Male | 71 | Obesity | 29.74 | 48 |
| PRJEB7774 | ERR688364 | Control | Austria | Male | 73 | Obesity | 29.7 | 48 |
| PRJEB7774 | ERR688374 | CRC | Austria | Male | 67 | Obesity | 29.4 | 49 |
| PRJEB7774 | ERR688373 | Control | Austria | Male | 65 | Obesity | 29.06 | 49 |
| PRJEB7774 | ERR688495 | CRC | Austria | Male | 66 | Normal | 22.6 | 50 |
| PRJEB7774 | ERR688376 | Control | Austria | Male | 67 | Normal | 22.79 | 50 |
| PRJEB7774 | ERR688642 | CRC | Austria | Male | 66 | Normal | 22.6 | 51 |
| PRJEB7774 | ERR688383 | Control | Austria | Male | 68 | Normal | 22.2 | 51 |
| PRJEB7774 | ERR688521 | CRC | Austria | Male | 67 | Obesity | 29.4 | 52 |
| PRJEB7774 | ERR688403 | Control | Austria | Male | 64 | Obesity | 29.3 | 52 |
| PRJEB7774 | ERR688358 | CRC | Austria | Male | 64 | Obesity | 29.35 | 53 |
| PRJEB7774 | ERR688404 | Control | Austria | Male | 65 | Obesity | 28.9 | 53 |
| PRJEB7774 | ERR688644 | CRC | Austria | Male | 65 | Obesity | 29.74 | 54 |
| PRJEB7774 | ERR688410 | Control | Austria | Male | 66 | Obesity | 29.98 | 54 |
| PRJEB7774 | ERR688428 | CRC | Austria | Female | 74 | Obesity | 29.17 | 55 |
| PRJEB7774 | ERR688412 | Control | Austria | Female | 72 | Obesity | 28.7 | 55 |
| PRJEB7774 | ERR688497 | CRC | Austria | Male | 65 | Obesity | 29.74 | 56 |
| PRJEB7774 | ERR688414 | Control | Austria | Male | 66 | Obesity | 30.02 | 56 |
| PRJEB7774 | ERR688500 | CRC | Austria | Male | 63 | Obesity | 33.56 | 57 |
| PRJEB7774 | ERR688418 | Control | Austria | Male | 66 | Obesity | 34.04 | 57 |
| PRJEB7774 | ERR688575 | CRC | Austria | Female | 74 | Obesity | 29.17 | 58 |
| PRJEB7774 | ERR688443 | Control | Austria | Female | 71 | Obesity | 29 | 58 |
| PRJEB7774 | ERR688505 | CRC | Austria | Male | 64 | Obesity | 29.35 | 59 |
| PRJEB7774 | ERR688444 | Control | Austria | Male | 64 | Obesity | 29.38 | 59 |
| PRJEB7774 | ERR688422 | CRC | Austria | Female | 73 | Obesity | 29.75 | 60 |
| PRJEB7774 | ERR688453 | Control | Austria | Female | 72 | Obesity | 30 | 60 |
| PRJEB7774 | ERR688494 | CRC | Austria | Male | 69 | Obesity | 29.94 | 61 |
| PRJEB7774 | ERR688477 | Control | Austria | Male | 68 | Obesity | 29.8 | 61 |
| PRJEB7774 | ERR688583 | CRC | Austria | Male | 43 | Normal | 22.68 | 62 |
| PRJEB7774 | ERR688499 | Control | Austria | Male | 46 | Normal | 23.15 | 62 |
| PRJEB7774 | ERR688401 | CRC | Austria | Male | 68 | Obesity | 32.25 | 63 |
| PRJEB7774 | ERR688506 | Control | Austria | Male | 68 | Obesity | 32 | 63 |
| PRJEB7774 | ERR688580 | CRC | Austria | Male | 71 | Obesity | 29.74 | 64 |
| PRJEB7774 | ERR688511 | Control | Austria | Male | 73 | Obesity | 29.7 | 64 |
| PRJEB7774 | ERR688639 | CRC | Austria | Male | 64 | Obesity | 29.07 | 65 |
| PRJEB7774 | ERR688520 | Control | Austria | Male | 65 | Obesity | 29.06 | 65 |
| PRJEB7774 | ERR688492 | CRC | Austria | Male | 64 | Obesity | 29.07 | 66 |
| PRJEB7774 | ERR688550 | Control | Austria | Male | 64 | Obesity | 29.3 | 66 |
| PRJEB7774 | ERR688647 | CRC | Austria | Male | 63 | Obesity | 33.56 | 67 |
| PRJEB7774 | ERR688565 | Control | Austria | Male | 66 | Obesity | 34.04 | 67 |
| PRJEB7774 | ERR688569 | CRC | Austria | Female | 73 | Obesity | 29.75 | 68 |
| PRJEB7774 | ERR688600 | Control | Austria | Female | 72 | Obesity | 30 | 68 |
| PRJEB7774 | ERR688436 | CRC | Austria | Male | 43 | Normal | 22.68 | 69 |
| PRJEB7774 | ERR688646 | Control | Austria | Male | 46 | Normal | 23.15 | 69 |
| PRJEB7774 | ERR688420 | CRC | Austria | Male | 54 | Overweight | 27.34 | 70 |
| PRJEB7774 | ERR710417 | Control | Austria | Male | 55 | Overweight | 27.76 | 70 |
| PRJEB7774 | ERR688425 | CRC | Austria | Male | 72 | Obesity | 28.41 | 71 |
| PRJEB7774 | ERR710418 | Control | Austria | Male | 70 | Obesity | 28.72 | 71 |
| PRJEB7774 | ERR688431 | CRC | Austria | Female | 72 | Overweight | 26.67 | 72 |
| PRJEB7774 | ERR710419 | Control | Austria | Female | 73 | Overweight | 26.56 | 72 |
| PRJEB7774 | ERR688502 | CRC | Austria | Female | 45 | Normal | 22.15 | 73 |
| PRJEB7774 | ERR710423 | Control | Austria | Female | 43 | Normal | 22.65 | 73 |
| PRJEB7774 | ERR688567 | CRC | Austria | Male | 54 | Overweight | 27.34 | 74 |
| PRJEB7774 | ERR710426 | Control | Austria | Male | 55 | Overweight | 27.76 | 74 |
| PRJEB7774 | ERR688572 | CRC | Austria | Male | 72 | Obesity | 28.41 | 75 |
| PRJEB7774 | ERR710427 | Control | Austria | Male | 70 | Obesity | 28.72 | 75 |
| PRJEB7774 | ERR688578 | CRC | Austria | Female | 72 | Overweight | 26.67 | 76 |
| PRJEB7774 | ERR710428 | Control | Austria | Female | 73 | Overweight | 26.56 | 76 |
| PRJEB7774 | ERR688649 | CRC | Austria | Female | 45 | Normal | 22.15 | 77 |
| PRJEB7774 | ERR710432 | Control | Austria | Female | 43 | Normal | 22.65 | 77 |
| PRJNA397219 | SRR5903342 | CRC | USA | Female | 78 | Overweight | 24.9 | 78 |
| PRJNA397219 | SRR5903321 | Control | USA | Female | 76 | Overweight | 25.28 | 78 |
| PRJNA397219 | SRR5903385 | CRC | USA | Female | 78 | Overweight | 24.9 | 79 |
| PRJNA397219 | SRR5903340 | Control | USA | Female | 76 | Overweight | 25.28 | 79 |
| PRJNA397219 | SRR5903368 | CRC | USA | Female | 78 | Overweight | 24.9 | 80 |
| PRJNA397219 | SRR5903347 | Control | USA | Female | 75 | Overweight | 25.4 | 80 |
| PRJNA447983 | SRR6915190 | CRC | Italy | Male | 78 | Normal | 24 | 81 |
| PRJNA447983 | SRR6915100 | Control | Italy | Male | 80 | Normal | 24 | 81 |
| PRJNA447983 | SRR6915188 | CRC | Italy | Male | 60 | Normal | 22 | 82 |
| PRJNA447983 | SRR6915145 | Control | Italy | Male | 60 | Normal | 22 | 82 |
| PRJNA447983 | SRR6915099 | CRC | Italy | Male | 63 | Overweight | 25 | 83 |
| PRJNA447983 | SRR6915187 | Control | Italy | Male | 64 | Overweight | 25 | 83 |
| PRJNA447983 | SRR6915119 | CRC | Italy | Male | 66 | Normal | 24 | 84 |
| PRJNA447983 | SRR6915191 | Control | Italy | Male | 69 | Normal | 24 | 84 |
| PRJNA447983 | SRR6915166 | CRC | Italy | Male | 70 | Normal | 24 | 85 |
| PRJNA447983 | SRR6915194 | Control | Italy | Male | 71 | Normal | 24 | 85 |
| PRJNA447983 | SRR6915210 | CRC | Italy | Male | 74 | Overweight | 25 | 86 |
| PRJNA447983 | SRR6915227 | Control | Italy | Male | 71 | Overweight | 25 | 86 |

**Table S2. The top 20 abundant species in the all paired-samples.**

| **Species** | **NCBI taxon id** | **Mean Relative Abundance (%)** | |
| --- | --- | --- | --- |
|  |  | **Control** | **CRC** |
| *Subdoligranulum sp* | 2053618 | 7.70 | 7.70 |
| *Faecalibacterium prausnitzii* | 853 | 8.57 | 6.15 |
| *Eubacterium rectale* | 39491 | 5.32 | 3.62 |
| *Ruminococcus bromii* | 40518 | 5.06 | 3.51 |
| *Bifidobacterium adolescentis* | 1680 | 3.96 | 4.41 |
| *Escherichia coli* | 562 | 2.90 | 4.19 |
| *Akkermansia muciniphila* | 239935 | 1.79 | 4.93 |
| *Prevotella copri* | 165179 | 3.66 | 2.71 |
| *Bacteroides uniformis* | 820 | 2.39 | 3.69 |
| *Alistipes putredinis* | 28117 | 2.24 | 2.76 |
| *Bifidobacterium longum* | 216816 | 2.88 | 1.88 |
| *Bacteroides vulgatus* | 821 | 1.96 | 2.25 |
| *Collinsella aerofaciens* | 74426 | 2.23 | 1.62 |
| *Ruminococcus torques* | 33039 | 1.65 | 2.02 |
| *Bacteroides stercoris* | 46506 | 2.27 | 1.16 |
| *Bacteroides dorei* | 357276 | 1.55 | 1.43 |
| *Megamonas sp.* | 2049033 | 1.56 | 0.98 |
| *Alistipes onderdonkii* | 328813 | 0.87 | 1.70 |
| *Eubacterium hallii* | 39488 | 1.28 | 1.20 |
| *Bacteroides eggerthii* | 28111 | 0.82 | 1.64 |
| *Others* | - | 39.33 | 40.46 |

**Notes:** The mean relative abundance represents the mean value of relative abundance of each species in each CRC and control group. The relative abundance represents the percentage of each species made of the organism per sample.

**Table S3 The top 20 abundant species in the CRC and controls distributed in six countries.**

| **Species, with**  **Mean Relative Abundance (%)** | **Australia** | | **China** | | **Germany** | | **Italy** | | **Japan** | | **USA** | |
| --- | --- | --- | --- | --- | --- | --- | --- | --- | --- | --- | --- | --- |
|  | **Control** | **CRC** | **Control** | **CRC** | **Control** | **CRC** | **Control** | **CRC** | **Control** | **CRC** | **Control** | **CRC** |
| *Subdoligranulum_sp* | 0.9526 | 0.9178 | 0.4646 | 0.5399 | 0.8951 | 0.9612 | 0.6692 | 0.9335 | 0.4134 | 0.6134 | 0.0000 | 0.0000 |
| *Faecalibacterium_prausnitzii* | 0.7019 | 0.6266 | 0.7616 | 0.4836 | 1.0183 | 1.0811 | 0.7306 | 0.9217 | 0.6836 | 0.5198 | 0.0972 | 0.2336 |
| *Eubacterium_rectale* | 0.5091 | 0.5534 | 0.5553 | 0.3090 | 0.8530 | 0.4755 | 0.2474 | 0.2333 | 0.5641 | 0.1598 | 0.1078 | 0.1092 |
| *Ruminococcus_bromii* | 0.6428 | 0.4350 | 0.4769 | 0.4105 | 0.3945 | 0.4247 | 0.0667 | 0.2414 | 0.0745 | 0.1889 | 0.9666 | 0.0000 |
| *Bifidobacterium_adolescentis* | 0.5829 | 0.4337 | 0.1485 | 0.3934 | 0.6162 | 0.6271 | 0.0463 | 0.0938 | 0.3131 | 0.0874 | 0.0000 | 0.0000 |
| *Escherichia_coli* | 0.2249 | 0.4262 | 0.4724 | 0.4478 | 0.2162 | 0.0587 | 0.2133 | 0.2577 | 0.1568 | 0.6867 | 0.2673 | 0.0706 |
| *Akkermansia_muciniphila* | 0.3463 | 0.3981 | 0.1070 | 0.2359 | 0.2338 | 0.3818 | 0.1186 | 0.4428 | 0.0422 | 0.1556 | 0.7191 | 0.8452 |
| *Prevotella_copri* | 0.0094 | 0.1132 | 0.1981 | 0.3748 | 0.0921 | 0.1141 | 0.6083 | 0.0197 | 0.3445 | 0.3255 | 0.0000 | 0.0000 |
| *Bacteroides_uniformis* | 0.2529 | 0.4095 | 0.3030 | 0.2646 | 0.5912 | 0.5405 | 0.4366 | 0.7728 | 0.3963 | 0.3434 | 0.3370 | 1.3803 |
| *Alistipes_putredinis* | 0.1743 | 0.2357 | 0.3841 | 0.3579 | 0.5363 | 0.5041 | 0.6381 | 0.6957 | 0.4026 | 0.0763 | 0.5938 | 1.0061 |
| *Bifidobacterium_longum* | 0.5349 | 0.4908 | 0.1831 | 0.2345 | 0.4313 | 0.2910 | 0.1339 | 0.2356 | 0.2937 | 0.1557 | 0.0122 | 0.0856 |
| *Bacteroides_vulgatus* | 0.2059 | 0.3464 | 0.3250 | 0.3066 | 0.4560 | 0.3506 | 0.4608 | 0.4478 | 0.4280 | 0.6020 | 0.2956 | 0.2818 |
| *Collinsella_aerofaciens* | 0.5336 | 0.3897 | 0.1514 | 0.3387 | 0.3751 | 0.2913 | 0.1354 | 0.0909 | 0.1797 | 0.1256 | 0.3392 | 0.0628 |
| *Ruminococcus_torques* | 0.3779 | 0.4046 | 0.2582 | 0.4460 | 0.2257 | 0.2698 | 0.2447 | 0.3049 | 0.2810 | 0.4927 | 0.8480 | 0.0390 |
| *Bacteroides_stercoris* | 0.0791 | 0.1323 | 0.3451 | 0.2310 | 0.1923 | 0.0547 | 0.1684 | 0.3241 | 0.5518 | 0.3320 | 0.0011 | 0.0000 |
| *Bacteroides_dorei* | 0.0693 | 0.1279 | 0.1588 | 0.2100 | 0.1841 | 0.2610 | 0.3053 | 0.2169 | 0.6650 | 0.1738 | 0.1427 | 0.0000 |
| *Megamonas_sp.* | 0.0055 | 0.0007 | 0.3805 | 0.3741 | 0.0000 | 0.0000 | 0.0000 | 0.0000 | 0.3363 | 0.2477 | 0.0000 | 0.0000 |
| *Alistipes_onderdonkii* | 0.1343 | 0.1349 | 0.0735 | 0.1436 | 0.2396 | 0.2533 | 0.3399 | 0.5644 | 0.0930 | 0.0411 | 0.3027 | 0.7623 |
| *Eubacterium_hallii* | 0.4568 | 0.3950 | 0.0689 | 0.1563 | 0.1663 | 0.0863 | 0.0088 | 0.0136 | 0.0459 | 0.1659 | 0.4039 | 0.0078 |
| *Bacteroides_eggerthii* | 0.0979 | 0.1722 | 0.2376 | 0.0735 | 0.0004 | 0.2937 | 0.0018 | 0.1955 | 0.0596 | 0.0036 | 0.0036 | 0.0000 |

**Notes:** The mean relative abundance represents the mean value of relative abundance of each species in each sub-regional group. The relative abundance represents the percentage of each species made of the organism per sample.

**Table S4 Species exclusively exists in the CRC groups**

| **NCBI taxon id** | **Species** | **Accumulated Abundance (%)** |
| --- | --- | --- |
| ncbi taxon id313439 | *Streptococcus massiliensis* | 0.02022 |
| ncbi taxon id199 | *Campylobacter concisus* | 0.02449 |
| ncbi taxon id1579 | *Lactobacillus acidophilus* | 0.0256 |
| ncbi taxon id626931 | *Bacteroides oleiciplenus* | 0.02878 |
| ncbi taxon id1280 | *Staphylococcus aureus* | 0.02908 |
| ncbi taxon id197614 | *Streptococcus pasteurianus* | 0.0314 |
| ncbi taxon id147802 | *Lactobacillus iners* | 0.03211 |
| ncbi taxon id58231 | *Treponema medium* | 0.03414 |
| ncbi taxon id419015 | *Alloscardovia omnicolens* | 0.03719 |
| ncbi taxon id837 | *Porphyromonas gingivalis* | 0.03763 |
| ncbi taxon id2049041 | *Slackia sp.* | 0.03833 |
| ncbi taxon id1872413 | *Aggregatibacter sp.* | 0.03901 |
| ncbi taxon id192066 | *Neisseria sp.* | 0.03942 |
| ncbi taxon id40214 | *Acinetobacter johnsonii* | 0.04087 |
| ncbi taxon id1078480 | *Haemophilus sputorum* | 0.04182 |
| ncbi taxon id1287640 | *Anaerococcus obesiensis* | 0.04224 |
| ncbi taxon id167 | *Treponema succinifaciens* | 0.04349 |
| ncbi taxon id437898 | *Sutterella parvirubra* | 0.04553 |
| ncbi taxon id61592 | *Corynebacterium durum* | 0.05056 |
| ncbi taxon id827 | *Campylobacter ureolyticus* | 0.0548 |
| ncbi taxon id54005 | *Peptoniphilus harei* | 0.05655 |
| ncbi taxon id1246 | *Leuconostoc lactis* | 0.0609 |
| ncbi taxon id28126 | *Prevotella buccae* | 0.06735 |
| ncbi taxon id102684 | *Streptococcus infantarius* | 0.06812 |
| ncbi taxon id469613 | *Enterobacteriaceae bacterium 9 2 54FAA* | 0.07208 |
| ncbi taxon id82135 | *Atopobium vaginae* | 0.07938 |
| ncbi taxon id1311 | *Streptococcus agalactiae* | 0.08149 |
| ncbi taxon id40324 | *Stenotrophomonas maltophilia* | 0.08307 |
| ncbi taxon id1930076 | *Leuconostoc sp.* | 0.0841 |
| ncbi taxon id1490 | *Paraclostridium bifermentans* | 0.09387 |
| ncbi taxon id1623 | *Lactobacillus ruminis* | 0.09538 |
| ncbi taxon id56774 | *Eubacterium infirmum* | 0.0976 |
| ncbi taxon id1747 | *Propionibacterium acnes* | 0.10179 |
| ncbi taxon id1077464 | *Streptococcus tigurinus* | 0.10209 |
| ncbi taxon id1381 | *Atopobium minutum* | 0.11521 |
| ncbi taxon id1338 | *Streptococcus intermedius* | 0.1241 |
| ncbi taxon id936375 | *Mogibacterium sp. CM50* | 0.13078 |
| ncbi taxon id33945 | *Enterococcus avium* | 0.14037 |
| ncbi taxon id1872471 | *Alloprevotella sp.* | 0.14389 |
| ncbi taxon id287 | *Pseudomonas aeruginosa* | 0.14871 |
| ncbi taxon id1492 | *Clostridium butyricum* | 0.15492 |
| ncbi taxon id76860 | *Streptococcus constellatus* | 0.15519 |
| ncbi taxon id28038 | *Lactobacillus curvatus* | 0.15863 |
| ncbi taxon id89153 | *Clostridium hylemonae* | 0.16513 |
| ncbi taxon id739 | *Aggregatibacter segnis* | 0.17538 |
| ncbi taxon id1334 | *Streptococcus dysgalactiae* | 0.17684 |
| ncbi taxon id860 | *Fusobacterium periodonticum* | 0.18781 |
| ncbi taxon id2042683 | *Olsenella sp.* | 0.19226 |
| ncbi taxon id177972 | *Shuttleworthia satelles* | 0.21087 |
| ncbi taxon id539 | *Eikenella corrodens* | 0.23557 |
| ncbi taxon id28129 | *Prevotella denticola* | 0.23753 |
| ncbi taxon id28124 | *Porphyromonas endodontalis* | 0.2522 |
| ncbi taxon id727 | *Haemophilus influenzae* | 0.30327 |
| ncbi taxon id78258 | *Parascardovia denticolens* | 0.32477 |
| ncbi taxon id28133 | *Prevotella nigrescens* | 0.33113 |
| ncbi taxon id33037 | *Anaerococcus vaginalis* | 0.33899 |
| ncbi taxon id47715 | *Lactobacillus rhamnosus* | 0.34071 |
| ncbi taxon id411484 | *Clostridium sp. SS2 1* | 0.34668 |
| ncbi taxon id104608 | *Leptotrichia sp.* | 0.34731 |
| ncbi taxon id638849 | *Pyramidobacter piscolens* | 0.37644 |
| ncbi taxon id584 | *Proteus mirabilis* | 0.38084 |
| ncbi taxon id726 | *Haemophilus haemolyticus* | 0.41415 |
| ncbi taxon id626938 | *Succinatimonas hippei* | 0.44008 |
| ncbi taxon id53345 | *Enterococcus durans* | 0.53291 |
| ncbi taxon id1226322 | *Oscillibacter sp. KLE 1728* | 0.54771 |
| ncbi taxon id84136 | *Gemella bergeri* | 0.55542 |
| ncbi taxon id103892 | *Veillonella ratti* | 0.59977 |
| ncbi taxon id1625 | *Lactobacillus sanfranciscensis* | 0.64683 |
| ncbi taxon id626930 | *Bacteroides fluxus* | 0.69636 |
| ncbi taxon id582 | *Morganella morganii* | 0.81539 |
| ncbi taxon id143361 | *Filifactor alocis* | 0.88227 |
| ncbi taxon id1598 | *Lactobacillus reuteri* | 0.92556 |
| ncbi taxon id1226325 | *Clostridium sp. KLE 1755* | 1.03666 |
| ncbi taxon id859 | *Fusobacterium necrophorum* | 1.09981 |
| ncbi taxon id469592 | *Bacteroides sp. 3 1 19* | 1.43444 |
| ncbi taxon id1261 | *Peptostreptococcus anaerobius* | 1.56898 |
| ncbi taxon id624 | *Shigella sonnei* | 1.7702 |
| ncbi taxon id52226 | *Mitsuokella multacida* | 1.90565 |
| ncbi taxon id97478 | *Lactobacillus mucosae* | 2.32566 |
| ncbi taxon id281920 | *Porphyromonas uenonis* | 2.55289 |
| ncbi taxon id28131 | *Prevotella intermedia* | 4.70568 |
| ncbi taxon id322095 | *Porphyromonas somerae* | 4.96671 |
| ncbi taxon id197 | *Campylobacter jejuni* | 7.87389 |
| ncbi taxon id626933 | *Odoribacter laneus* | 8.59283 |
| ncbi taxon id626935 | *Collinsella tanakaei* | 10.83231 |
| ncbi taxon id457415 | *Synergistes sp. 3 1 syn1* | 12.60717 |
| ncbi taxon id457394 | *Bacteroides sp. 4 3 47FAA* | 17.49437 |

**Table S5 Species exclusively exists in the Control groups**

| **ncbi taxon id** | **Species** | **Acumulated**  **Abundance (%)** |
| --- | --- | --- |
| ncbi taxon id1281 | *Staphylococcus carnosus* | 0.02264 |
| ncbi taxon id1423 | *Bacillus subtilis* | 0.02576 |
| ncbi taxon id1260 | *Finegoldia magna* | 0.02646 |
| ncbi taxon id33031 | *Peptoniphilus lacrimalis* | 0.03035 |
| ncbi taxon id133926 | *Olsenella uli* | 0.03109 |
| ncbi taxon id1604 | *Lactobacillus amylovorus* | 0.03136 |
| ncbi taxon id484 | *Neisseria flavescens* | 0.03456 |
| ncbi taxon id1244 | *Leuconostoc gelidum* | 0.03533 |
| ncbi taxon id1891233 | *Dysgonomonas sp.* | 0.04246 |
| ncbi taxon id60133 | *Prevotella pallens* | 0.04289 |
| ncbi taxon id539813 | *Enterobacter mori* | 0.04569 |
| ncbi taxon id614 | *Serratia liquefaciens* | 0.04598 |
| ncbi taxon id736 | *Haemophilus paraphrohaemolyticus* | 0.0555 |
| ncbi taxon id1590 | *Lactobacillus plantarum* | 0.05897 |
| ncbi taxon id1264 | *Ruminococcus albus* | 0.06673 |
| ncbi taxon id29385 | *Staphylococcus saprophyticus* | 0.08224 |
| ncbi taxon id552396 | *Erysipelotrichaceae bacterium 5 2 54FAA* | 0.13097 |
| ncbi taxon id54291 | *Raoultella ornithinolytica* | 0.23351 |
| ncbi taxon id742723 | *Lachnospiraceae bacterium 2 1 46FAA* | 0.23439 |
| ncbi taxon id1896336 | *Citrobacter sp.* | 0.28217 |
| ncbi taxon id824 | *Campylobacter gracilis* | 0.42878 |
| ncbi taxon id1161942 | *Ruminococcus champanellensis* | 0.55165 |
| ncbi taxon id469585 | *Bacteroides sp. 1 1 14* | 0.77844 |
| ncbi taxon id36773 | *Burkholderia sp.* | 0.78097 |
| ncbi taxon id1633 | *Lactobacillus vaginalis* | 1.10698 |
| ncbi taxon id188913 | *Cetobacterium somerae* | 2.36553 |
| ncbi taxon id41978 | *Ruminococcus sp.* | 2.97632 |
| ncbi taxon id1683 | *Bifidobacterium angulatum* | 5.12335 |
| ncbi taxon id384638 | *Bacteroides pectinophilus* | 20.08122 |
| ncbi taxon id1605 | *Lactobacillus animalis* | 26.2263 |

**Table S6. Species significantly different in abundance in the CRC and healthy patients (with |LogFC|＞1.0 and *p<0.05*)**

| **Taxon ID** | **Species_name** | **LogFC** | ***p_value*** | ***FDR*** | **Trend**  **(CRC v.s Health)** |
| --- | --- | --- | --- | --- | --- |
| 28131 | Prevotella_intermedia | -15.26 | 0.01 | 0.21 | depleted |
| 1625 | Lactobacillus_sanfranciscensis | -12.40 | 0.01 | 0.19 | depleted |
| 1261 | Peptostreptococcus_anaerobius | -11.87 | 0.03 | 0.39 | depleted |
| 104608 | Leptotrichia_sp | -11.50 | 0.04 | 0.39 | depleted |
| 28123 | Porphyromonas_asaccharolytica | -10.68 | 0.00 | 0.01 | depleted |
| 626935 | Collinsella_tanakaei | -9.31 | 0.00 | 0.03 | depleted |
| 53345 | Enterococcus_durans | -8.74 | 0.03 | 0.39 | depleted |
| 322095 | Porphyromonas_somerae | -8.52 | 0.01 | 0.19 | depleted |
| 281920 | Porphyromonas_uenonis | -7.19 | 0.00 | 0.08 | depleted |
| 1686 | Bifidobacterium_catenulatum | 6.90 | 0.01 | 0.19 | enriched |
| 76860 | Streptococcus_constellatus | -6.72 | 0.01 | 0.21 | depleted |
| 33033 | Parvimonas_micra | -6.43 | 0.00 | 0.00 | depleted |
| 29391 | Gemella_morbillorum | -6.40 | 0.00 | 0.02 | depleted |
| 1584 | Lactobacillus_delbrueckii | 6.16 | 0.04 | 0.42 | enriched |
| 1944660 | Parvimonas_sp | -6.02 | 0.00 | 0.00 | depleted |
| 1736 | Eubacterium_limosum | -5.90 | 0.00 | 0.03 | depleted |
| 851 | Fusobacterium_nucleatum | -5.56 | 0.00 | 0.03 | depleted |
| 89153 | Clostridium_hylemonae | -5.53 | 0.01 | 0.19 | depleted |
| 1338 | Streptococcus_intermedius | -5.52 | 0.02 | 0.30 | depleted |
| 341694 | Peptostreptococcus_stomatis | -5.18 | 0.00 | 0.01 | depleted |
| 2042683 | Olsenella_sp | -5.13 | 0.00 | 0.06 | depleted |
| 1582 | Lactobacillus_casei | 5.04 | 0.00 | 0.08 | enriched |
| 100886 | Catenibacterium_mitsuokai | -4.82 | 0.00 | 0.03 | depleted |
| 363265 | Prevotella_stercorea | -4.67 | 0.00 | 0.13 | depleted |
| 1077464 | Streptococcus_tigurinus | -4.17 | 0.01 | 0.21 | depleted |
| 1896336 | Citrobacter_sp | 3.94 | 0.04 | 0.39 | enriched |
| 46124 | Granulicatella_adiacens | -3.44 | 0.00 | 0.02 | depleted |
| 1262 | Peptostreptococcus_sp | -3.22 | 0.01 | 0.19 | depleted |
| 45634 | Streptococcus_cristatus | -3.14 | 0.02 | 0.28 | depleted |
| 102148 | Solobacterium_moorei | -3.06 | 0.00 | 0.03 | depleted |
| 1328 | Streptococcus_anginosus | -2.59 | 0.01 | 0.19 | depleted |
| 626932 | Alistipes_indistinctus | -2.56 | 0.00 | 0.06 | depleted |
| 1343 | Streptococcus_vestibularis | -2.34 | 0.02 | 0.29 | depleted |
| 66852 | Methanobrevibacter_sp | -2.27 | 0.02 | 0.22 | depleted |
| 28037 | Streptococcus_mitis | -1.94 | 0.01 | 0.19 | depleted |
| 328812 | Parabacteroides_goldsteinii | -1.80 | 0.04 | 0.39 | depleted |
| 665956 | Subdoligranulum_sp_4_3_54A2FAA | -1.73 | 0.03 | 0.34 | depleted |
| 2173 | Methanobrevibacter_smithii | -1.70 | 0.01 | 0.19 | depleted |
| 39778 | Veillonella_dispar | -1.67 | 0.05 | 0.46 | depleted |
| 39496 | Eubacterium_ventriosum | 1.54 | 0.00 | 0.06 | enriched |
| 239935 | Akkermansia_muciniphila | -1.46 | 0.01 | 0.21 | depleted |
| 1872531 | Anaerotruncus_sp | -1.43 | 0.04 | 0.39 | depleted |
| 214856 | Alistipes_finegoldii | -1.30 | 0.02 | 0.27 | depleted |
| 1302 | Streptococcus_gordonii | -1.27 | 0.02 | 0.22 | depleted |

**Table S7. The top 50 significant species identified in the Random Forest Model**

| **Species** | **Taxon ID** | **Mean Decrease Accuracy** | **p-value** |
| --- | --- | --- | --- |
| *Parvimonas micra* | 33033 | 0.0039 | 0.0000 |
| *Porphyromonas asaccharolytica* | 28123 | 0.0036 | 0.0000 |
| *Catenibacterium mitsuokai* | 100886 | 0.0035 | 0.0001 |
| *Peptostreptococcus stomatis* | 341694 | 0.0033 | 0.0000 |
| *Parvimonas sp.* | 1944660 | 0.0023 | 0.0000 |
| *Ruminococcus callidus* | 40519 | 0.0018 | 0.0424 |
| *Eubacterium rectale* | 39491 | 0.0017 | 0.1162 |
| *Solobacterium moorei* | 102148 | 0.0016 | 0.0031 |
| *Bacteroides ovatus* | 28116 | 0.0015 | 0.3490 |
| *Fusobacterium nucleatum* | 851 | 0.0014 | 0.0006 |
| *Alistipes putredinis* | 28117 | 0.0014 | 0.6871 |
| *Alistipes communis* | 2585118 | 0.0013 | 0.5862 |
| *Parabacteroides goldsteinii* | 328812 | 0.0013 | 0.0020 |
| *Roseburia intestinalis* | 166486 | 0.0012 | 0.0193 |
| *Alistipes finegoldii* | 214856 | 0.0011 | 0.1847 |
| *Prevotella stercorea* | 363265 | 0.0011 | 0.0025 |
| *Olsenella sp.* | 2042683 | 0.0011 | 0.0025 |
| *Lachnospiraceae bacterium 2 1 58FAA* | 658082 | 0.0011 | 0.0939 |
| *Bilophila sp.* | 1929485 | 0.0011 | 0.8332 |
| *Ruminococcus torques* | 33039 | 0.0010 | 0.3175 |
| *Bacteroides thetaiotaomicron* | 818 | 0.0010 | 0.5749 |
| *Alistipes onderdonkii* | 328813 | 0.0010 | 0.0434 |
| *Clostridium leptum* | 1535 | 0.0010 | 0.0925 |
| *Collinsella tanakaei* | 626935 | 0.0010 | 0.0052 |
| *Subdoligranulum sp* | 2053618 | 0.0009 | 0.3295 |
| *Clostridium asparagiforme* | 333367 | 0.0009 | 0.4424 |
| *Bacteroides uniformis* | 820 | 0.0009 | 0.0387 |
| *Gemella morbillorum* | 29391 | 0.0008 | 0.0000 |
| *Prevotella copri* | 165179 | 0.0008 | 0.1681 |
| *Lachnospiraceae bacterium 5 1 63FAA* | 658089 | 0.0008 | 0.2447 |
| *Bifidobacterium bifidum* | 1681 | 0.0008 | 0.5620 |
| *Intestinibacter bartlettii* | 261299 | 0.0008 | 0.1983 |
| *Eubacterium ramulus* | 39490 | 0.0008 | 0.1691 |
| *Ruminococcus gnavus* | 33038 | 0.0008 | 0.2879 |
| *Bacteroides vulgatus* | 821 | 0.0008 | 0.3211 |
| *Streptococcus sanguinis* | 1305 | 0.0008 | 0.0160 |
| *Lactobacillus casei* | 1582 | 0.0007 | 0.0099 |
| *Pseudoflavonifractor capillosus* | 106588 | 0.0007 | 0.0095 |
| *Methanobrevibacter smithii* | 2173 | 0.0007 | 0.0017 |
| *Coprococcus comes* | 410072 | 0.0006 | 0.3293 |
| *Bacteroides salyersiae* | 291644 | 0.0006 | 0.3337 |
| *Veillonella parvula* | 29466 | 0.0006 | 0.6792 |
| *Eubacterium eligens* | 39485 | 0.0006 | 0.2777 |
| *Erysipelatoclostridium ramosum* | 1547 | 0.0006 | 0.5639 |
| *Lachnospiraceae bacterium 3 1 57FAA CT1* | 658086 | 0.0006 | 0.0154 |
| *Clostridium symbiosum* | 1512 | 0.0006 | 0.3512 |
| *Bacteroides faecis* | 674529 | 0.0006 | 0.1919 |
| *Methanobrevibacter sp.* | 66852 | 0.0006 | 0.0266 |
| *Flavonifractor plautii* | 292800 | 0.0005 | 0.7062 |
| *Lactobacillus sanfranciscensis* | 1625 | 0.0005 | 0.0055 |

**Table S8. The performance of prediction models by integrating different numbers of microbe species from the top 11 to 50 microbes.**

|  | **Performance**  **(Median, 95% CI)** | **Models from the top 11-29 microbes** | **Models from the top 30-50 microbes** | ***p-value*** |
| --- | --- | --- | --- | --- |
| Training Cohort | AUC | 0.784 (0.575-0.816) | 0.804 (0.786-0.839) | *<0.001** |
|  | Sensitivity | 0.857 (0.778-0.882) | 0.905 (0.857-0.937) | *<0.001** |
|  | Specificity | 0.742 (0.655-0.774) | 0.742 (0.726-0.774) | *0.327* |
| Validation Cohort | AUC | 0.749 (0.692-0.775) | 0.800 (0.790-0.823) | *<0.001** |
|  | Sensitivity | 0.769 (0.742-0.857) | 0.905 (0.841-0.945) | *0.003** |
|  | Specificity | 0.677 (0.615-0.792) | 0.710 (0.694-0.762) | *0.242* |

Note: †p value was derived from the Wilcoxon Mann-Whitney test in data of continuous variables with abnormal distribution.

**Table S9. Performance of prediction models by integrating different numbers of microbe species from the top 11 to 50 microbes.**

| **Number of Variables** | **AUC_T** | **Sens_T** | **Spec_T** | **AUC_V** | **Sens_V** | **Spec_V** |
| --- | --- | --- | --- | --- | --- | --- |
| 11 | 0.680 | 0.739 | 0.621 | 0.646 | 0.750 | 0.542 |
| 12 | 0.726 | 0.852 | 0.600 | 0.698 | 0.864 | 0.533 |
| 13 | 0.769 | 0.778 | 0.760 | 0.692 | 0.769 | 0.615 |
| 14 | 0.752 | 0.800 | 0.704 | 0.772 | 0.840 | 0.704 |
| 15 | 0.757 | 0.862 | 0.652 | 0.704 | 0.742 | 0.667 |
| 16 | 0.767 | 0.815 | 0.720 | 0.696 | 0.800 | 0.593 |
| 17 | 0.769 | 0.778 | 0.760 | 0.697 | 0.594 | 0.800 |
| 18 | 0.714 | 0.700 | 0.727 | 0.682 | 0.571 | 0.792 |
| 19 | 0.816 | 0.857 | 0.774 | 0.768 | 0.774 | 0.762 |
| 20 | 0.824 | 0.964 | 0.583 | 0.822 | 0.917 | 0.590 |
| 21 | 0.816 | 0.857 | 0.774 | 0.792 | 0.767 | 0.818 |
| 22 | 0.832 | 0.857 | 0.806 | 0.692 | 0.769 | 0.615 |
| 23 | 0.816 | 0.857 | 0.774 | 0.749 | 0.759 | 0.739 |
| 24 | 0.784 | 0.719 | 0.850 | 0.775 | 0.905 | 0.645 |
| 25 | 0.798 | 0.882 | 0.714 | 0.791 | 0.905 | 0.677 |
| 26 | 0.800 | 0.857 | 0.742 | 0.767 | 0.857 | 0.677 |
| 27 | 0.800 | 0.857 | 0.742 | 0.673 | 0.679 | 0.667 |
| 28 | 0.762 | 0.870 | 0.655 | 0.758 | 0.690 | 0.826 |
| 29 | 0.800 | 0.857 | 0.742 | 0.814 | 0.759 | 0.870 |
| 30 | 0.887 | 0.946 | 0.708 | 0.788 | 0.850 | 0.590 |
| 31 | 0.839 | 0.905 | 0.774 | 0.792 | 0.833 | 0.750 |
| 32 | 0.839 | 0.905 | 0.774 | 0.783 | 0.708 | 0.710 |
| 33 | 0.856 | 0.905 | 0.806 | 0.839 | 0.905 | 0.774 |
| 34 | 0.839 | 0.905 | 0.774 | 0.823 | 0.905 | 0.742 |
| 35 | 0.823 | 0.905 | 0.742 | 0.783 | 0.857 | 0.710 |
| 36 | 0.839 | 0.905 | 0.774 | 0.791 | 0.905 | 0.677 |
| 37 | 0.816 | 0.857 | 0.774 | 0.808 | 0.810 | 0.806 |
| 38 | 0.800 | 0.857 | 0.742 | 0.807 | 0.905 | 0.710 |
| 39 | 0.816 | 0.857 | 0.774 | 0.783 | 0.708 | 0.710 |
| 40 | 0.865 | 0.857 | 0.708 | 0.796 | 0.883 | 0.623 |
| 41 | 0.823 | 0.905 | 0.742 | 0.783 | 0.857 | 0.710 |
| 42 | 0.800 | 0.857 | 0.742 | 0.823 | 0.905 | 0.742 |
| 43 | 0.823 | 0.905 | 0.742 | 0.791 | 0.905 | 0.677 |
| 44 | 0.783 | 0.857 | 0.710 | 0.792 | 0.810 | 0.774 |
| 45 | 0.807 | 0.905 | 0.710 | 0.839 | 0.905 | 0.774 |
| 46 | 0.823 | 0.905 | 0.742 | 0.807 | 0.905 | 0.710 |
| 47 | 0.783 | 0.857 | 0.710 | 0.800 | 0.857 | 0.742 |
| 48 | 0.823 | 0.905 | 0.742 | 0.807 | 0.905 | 0.710 |
| 49 | 0.800 | 0.857 | 0.742 | 0.839 | 0.905 | 0.774 |
| 50 | 0.857 | 0.952 | 0.742 | 0.846 | 0.950 | 0.639 |

Notes: AUC, Area under the curves; T: training cohort; Sens: sensitivity; Spec: specificity; V: validation cohort.

**Table S10. Correlation coefficients (Rho) and p values for the Spearmen correlation of different species and age/BMI.**

| ***Species*** | **Rho (BMI)** | ***p-value (BMI)*** | **Rho (Age)** | ***p-value (Age)*** |
| --- | --- | --- | --- | --- |
| *Adlercreutzia_equolifaciens* | 0.417 | <0.001 | -0.029 | 0.857 |
| *Eubacterium_hallii* | 0.385 | <0.001 | 0.004 | 0.980 |
| *Blautia_obeum* | 0.376 | <0.001 | -0.054 | 0.720 |
| *Dorea_longicatena* | 0.303 | 0.001 | 0.026 | 0.873 |
| *Collinsella_aerofaciens* | 0.250 | 0.012 | 0.077 | 0.592 |
| *Propionibacterium_freudenreichii* | 0.212 | 0.041 | 0.170 | 0.127 |
| *Eubacterium_rectale* | 0.211 | 0.042 | 0.001 | 0.993 |
| *Bifidobacterium_adolescentis* | 0.210 | 0.044 | -0.162 | 0.150 |
| *Coprococcus_catus* | 0.200 | 0.059 | -0.055 | 0.718 |
| *Subdoligranulum_sp* | 0.197 | 0.063 | -0.104 | 0.427 |
| *Akkermansia_muciniphila* | 0.195 | 0.067 | 0.039 | 0.805 |
| *Turicibacter_sanguinis* | 0.195 | 0.067 | 0.271 | 0.005 |
| *Bacteroides_pectinophilus* | 0.190 | 0.076 | 0.112 | 0.379 |
| *Turicibacter_sp.* | 0.186 | 0.086 | 0.250 | 0.011 |
| *Anaerostipes_hadrus* | 0.175 | 0.111 | 0.021 | 0.897 |
| *Bifidobacterium_longum* | 0.175 | 0.112 | -0.140 | 0.239 |
| *Coprococcus_eutactus* | 0.169 | 0.129 | 0.084 | 0.545 |
| *Bifidobacterium_animalis* | 0.154 | 0.179 | 0.099 | 0.459 |
| *Lactobacillus_delbrueckii* | 0.149 | 0.199 | 0.054 | 0.721 |
| *Slackia_piriformis* | 0.141 | 0.233 | 0.024 | 0.883 |
| *Parabacteroides_sp._D13* | 0.120 | 0.333 | 0.050 | 0.743 |
| *Streptococcus_thermophilus* | 0.116 | 0.359 | -0.025 | 0.878 |
| *Lactobacillus_salivarius* | 0.114 | 0.369 | 0.106 | 0.413 |
| *Ruminococcus_sp.* | 0.113 | 0.376 | 0.102 | 0.439 |
| *Roseburia_sp.* | 0.111 | 0.388 | -0.063 | 0.673 |
| *Lactobacillus_animalis* | 0.109 | 0.401 | 0.046 | 0.766 |
| *Coprococcus_comes* | 0.096 | 0.476 | -0.271 | 0.005 |
| *Porphyromonas_asaccharolytica* | 0.095 | 0.481 | 0.002 | 0.988 |
| *Lactobacillus_fermentum* | 0.095 | 0.483 | 0.055 | 0.713 |
| *Ruminococcus_lactaris* | 0.092 | 0.502 | -0.158 | 0.164 |
| *Ruminococcus_torques* | 0.091 | 0.507 | -0.031 | 0.846 |
| *Bifidobacterium_angulatum* | 0.090 | 0.514 | -0.074 | 0.605 |
| *Coprobacillus_cateniformis* | 0.088 | 0.523 | 0.178 | 0.104 |
| *Methanobrevibacter_smithii* | 0.081 | 0.564 | 0.040 | 0.800 |
| *Lactococcus_lactis* | 0.074 | 0.609 | -0.090 | 0.513 |
| *Dorea_formicigenerans* | 0.073 | 0.615 | -0.134 | 0.267 |
| *Porphyromonas_uenonis* | 0.072 | 0.620 | 0.034 | 0.830 |
| *Lachnospiraceae_bacterium_5_1_63FAA* | 0.070 | 0.628 | -0.067 | 0.651 |
| *Coprobacter_fastidiosus* | 0.070 | 0.633 | -0.110 | 0.395 |
| *Ruminococcus_bromii* | 0.067 | 0.650 | -0.074 | 0.609 |
| *Methanobrevibacter_sp.* | 0.064 | 0.664 | 0.067 | 0.648 |
| *Phascolarctobacterium_succinatutens* | 0.063 | 0.674 | 0.087 | 0.529 |
| *Ruminococcus_sp._5_1_39BFAA* | 0.062 | 0.677 | -0.128 | 0.294 |
| *Eubacterium_siraeum* | 0.062 | 0.677 | 0.094 | 0.485 |
| *Ruminococcus_sp._JC304* | 0.060 | 0.689 | -0.055 | 0.716 |
| *Porphyromonas_somerae* | 0.059 | 0.694 | -0.024 | 0.882 |
| *Clostridium_spiroforme* | 0.058 | 0.698 | 0.136 | 0.255 |
| *Dialister_invisus* | 0.056 | 0.709 | -0.010 | 0.950 |
| *Lactobacillus_casei* | 0.051 | 0.737 | 0.055 | 0.716 |
| *Eubacterium_sp._3_1_31* | 0.051 | 0.738 | 0.104 | 0.428 |
| *Gordonibacter_pamelaeae* | 0.048 | 0.755 | 0.069 | 0.639 |
| *Holdemanella_biformis* | 0.046 | 0.765 | -0.057 | 0.702 |
| *Butyrivibrio_crossotus* | 0.042 | 0.787 | 0.069 | 0.640 |
| *Eubacterium_ramulus* | 0.040 | 0.799 | 0.014 | 0.927 |
| *Bacteroides_salanitronis* | 0.036 | 0.818 | -0.059 | 0.694 |
| *Bifidobacterium_bifidum* | 0.033 | 0.833 | 0.084 | 0.545 |
| *Enterococcus_faecium* | 0.032 | 0.841 | -0.049 | 0.749 |
| *Intestinibacter_bartlettii* | 0.031 | 0.844 | -0.025 | 0.879 |
| *Klebsiella_sp.* | 0.024 | 0.882 | 0.014 | 0.928 |
| *Alistipes_sp._HGB5* | 0.019 | 0.905 | 0.053 | 0.727 |
| *Alistipes_onderdonkii* | 0.009 | 0.957 | -0.033 | 0.832 |
| *Odoribacter_laneus* | 0.006 | 0.970 | 0.038 | 0.807 |
| *Lachnospiraceae_bacterium_1_4_56FAA* | 0.005 | 0.973 | 0.179 | 0.103 |
| *Eubacterium_ventriosum* | 0.002 | 0.992 | -0.126 | 0.305 |
| *Holdemania_filiformis* | 0.001 | 0.992 | 0.051 | 0.740 |
| *Barnesiella_intestinihominis* | 0.000 | 0.998 | -0.047 | 0.756 |
| *Bifidobacterium_dentium* | -0.001 | 0.993 | 0.251 | 0.011 |
| *Klebsiella_oxytoca* | -0.006 | 0.972 | -0.016 | 0.921 |
| *Alistipes_obesi* | -0.006 | 0.970 | 0.301 | 0.001 |
| *unclassified_Peptostreptococcaceae__miscellaneous_* | -0.007 | 0.964 | 0.079 | 0.579 |
| *Faecalibacterium_prausnitzii* | -0.008 | 0.959 | -0.068 | 0.641 |
| *Roseburia_hominis* | -0.010 | 0.951 | 0.054 | 0.720 |
| *Lachnospiraceae_bacterium_8_1_57FAA* | -0.010 | 0.948 | -0.125 | 0.311 |
| *Faecalitalea_cylindroides* | -0.012 | 0.940 | -0.044 | 0.779 |
| *Anaerotruncus_sp.* | -0.014 | 0.932 | -0.041 | 0.790 |
| *Alistipes_putredinis* | -0.018 | 0.911 | -0.086 | 0.537 |
| *Ruminococcus_callidus* | -0.019 | 0.908 | -0.030 | 0.849 |
| *Oscillibacter_sp._KLE_1745* | -0.020 | 0.901 | 0.055 | 0.715 |
| *Prevotella_intermedia* | -0.023 | 0.886 | 0.084 | 0.545 |
| *Escherichia_sp.* | -0.025 | 0.875 | -0.060 | 0.690 |
| *Tyzzerella_nexilis* | -0.030 | 0.852 | 0.006 | 0.971 |
| *Dorea_sp.* | -0.033 | 0.835 | -0.124 | 0.314 |
| *Clostridium_sp._L250* | -0.037 | 0.814 | -0.013 | 0.937 |
| *Streptococcus_sanguinis* | -0.038 | 0.807 | -0.025 | 0.878 |
| *Synergistes_sp._3_1_syn1* | -0.038 | 0.807 | 0.096 | 0.475 |
| *Eggerthella_lenta* | -0.042 | 0.789 | 0.015 | 0.927 |
| *Lachnospiraceae_bacterium_3_1_46FAA* | -0.049 | 0.750 | -0.012 | 0.941 |
| *Bifidobacterium_catenulatum* | -0.050 | 0.745 | -0.109 | 0.397 |
| *Streptococcus_vestibularis* | -0.052 | 0.734 | 0.089 | 0.519 |
| *Lachnospiraceae_bacterium_3_1_57FAA_CT1* | -0.052 | 0.729 | -0.034 | 0.832 |
| *Bacteroides_coprophilus* | -0.055 | 0.716 | 0.040 | 0.800 |
| *Catenibacterium_mitsuokai* | -0.056 | 0.712 | -0.071 | 0.625 |
| *Streptococcus_salivarius* | -0.056 | 0.707 | -0.009 | 0.958 |
| *Clostridium_sp._ATCC_BAA442* | -0.058 | 0.699 | -0.047 | 0.758 |
| *Mitsuokella_multacida* | -0.058 | 0.698 | -0.043 | 0.781 |
| *Coprococcus_sp._ART55_1* | -0.061 | 0.681 | -0.124 | 0.314 |
| *Bacteroides_eggerthii* | -0.065 | 0.661 | 0.005 | 0.977 |
| *Lactobacillus_mucosae* | -0.065 | 0.659 | -0.055 | 0.714 |
| *Burkholderiales_bacterium_1_1_47* | -0.067 | 0.647 | 0.056 | 0.712 |
| *Clostridium_leptum* | -0.071 | 0.627 | -0.027 | 0.864 |
| *Acidaminococcus_sp.* | -0.071 | 0.624 | -0.040 | 0.797 |
| *Alistipes_finegoldii* | -0.073 | 0.616 | 0.004 | 0.980 |
| *Paraprevotella_sp* | -0.074 | 0.610 | -0.118 | 0.347 |
| *Bacteroides_faecis* | -0.074 | 0.609 | -0.059 | 0.695 |
| *Dialister_succinatiphilus* | -0.075 | 0.600 | -0.052 | 0.732 |
| *Paraprevotella_clara* | -0.077 | 0.588 | -0.006 | 0.972 |
| *Oxalobacter_formigenes* | -0.081 | 0.565 | -0.141 | 0.230 |
| *Blautia_hydrogenotrophica* | -0.082 | 0.560 | 0.003 | 0.986 |
| *Subdoligranulum_sp._4_3_54A2FAA* | -0.082 | 0.558 | 0.048 | 0.755 |
| *Ruminococcaceae_bacterium_D16* | -0.085 | 0.540 | -0.004 | 0.981 |
| *Alistipes_communis* | -0.091 | 0.503 | -0.054 | 0.719 |
| *Alistipes_sp.* | -0.092 | 0.500 | 0.067 | 0.651 |
| *Gemella_morbillorum* | -0.097 | 0.469 | -0.086 | 0.539 |
| *Peptostreptococcus_stomatis* | -0.101 | 0.445 | 0.010 | 0.949 |
| *Bacteroides_coprocola* | -0.107 | 0.412 | -0.130 | 0.283 |
| *Collinsella_intestinalis* | -0.107 | 0.410 | -0.227 | 0.026 |
| *Parasutterella_excrementihominis* | -0.109 | 0.397 | 0.003 | 0.985 |
| *Escherichia_coli* | -0.110 | 0.394 | 0.015 | 0.923 |
| *Prevotella_copri* | -0.117 | 0.352 | -0.054 | 0.722 |
| *Candidatus_Alistipes_marseilloanorexicus_AP11* | -0.120 | 0.336 | -0.011 | 0.944 |
| *Cetobacterium_somerae* | -0.122 | 0.322 | -0.052 | 0.729 |
| *Bacteroides_plebeius* | -0.123 | 0.320 | -0.038 | 0.807 |
| *Bacteroides_sp._2_1_22* | -0.123 | 0.318 | -0.104 | 0.429 |
| *Bacteroides_sp._4_3_47FAA* | -0.129 | 0.290 | -0.002 | 0.990 |
| *Odoribacter_splanchnicus* | -0.129 | 0.290 | 0.089 | 0.516 |
| *Bacteroides_uniformis* | -0.130 | 0.283 | -0.026 | 0.873 |
| *Oscillibacter_sp.* | -0.142 | 0.228 | -0.111 | 0.386 |
| *Desulfovibrio_piger* | -0.142 | 0.226 | -0.088 | 0.527 |
| *Anaerostipes_caccae* | -0.142 | 0.226 | -0.043 | 0.781 |
| *Parvimonas_sp.* | -0.147 | 0.207 | -0.064 | 0.667 |
| *Bacteroides_massiliensis* | -0.148 | 0.203 | -0.187 | 0.083 |
| *Eubacterium_eligens* | -0.148 | 0.201 | -0.030 | 0.852 |
| *Lachnospiraceae_bacterium_1_1_57FAA* | -0.149 | 0.200 | -0.073 | 0.616 |
| *Parabacteroides_johnsonii* | -0.150 | 0.195 | -0.062 | 0.679 |
| *Eggerthella_sp.* | -0.151 | 0.191 | -0.119 | 0.343 |
| *Ruminococcus_gnavus* | -0.151 | 0.190 | -0.008 | 0.962 |
| *Alistipes_shahii* | -0.153 | 0.183 | -0.222 | 0.030 |
| *Desulfovibrio_desulfuricans* | -0.158 | 0.166 | 0.015 | 0.923 |
| *Streptococcus_parasanguinis* | -0.163 | 0.149 | 0.007 | 0.966 |
| *Bacteroides_salyersiae* | -0.164 | 0.143 | 0.014 | 0.928 |
| *Campylobacter_jejuni* | -0.168 | 0.131 | -0.074 | 0.605 |
| *Parvimonas_micra* | -0.169 | 0.129 | 0.007 | 0.967 |
| *Prevotella_stercorea* | -0.170 | 0.126 | 0.087 | 0.528 |
| *Bifidobacterium_pseudocatenulatum* | -0.173 | 0.118 | -0.277 | 0.004 |
| *Clostridium_asparagiforme* | -0.176 | 0.109 | 0.078 | 0.584 |
| *Roseburia_inulinivorans* | -0.177 | 0.107 | -0.184 | 0.091 |
| *Erysipelatoclostridium_ramosum* | -0.181 | 0.098 | 0.010 | 0.949 |
| *Alistipes_indistinctus* | -0.182 | 0.094 | -0.082 | 0.558 |
| *Roseburia_intestinalis* | -0.183 | 0.092 | -0.078 | 0.587 |
| *Alistipes_senegalensis* | -0.185 | 0.087 | -0.041 | 0.791 |
| *Collinsella_tanakaei* | -0.186 | 0.086 | 0.061 | 0.681 |
| *Fusobacterium聽mortiferum* | -0.188 | 0.081 | -0.027 | 0.868 |
| *Solobacterium_moorei* | -0.191 | 0.075 | -0.089 | 0.520 |
| *Anaerotruncus_colihominis* | -0.191 | 0.075 | 0.034 | 0.829 |
| *Lachnospiraceae_bacterium_7_1_58FAA* | -0.191 | 0.074 | 0.017 | 0.917 |
| *Anaerostipes_sp.* | -0.196 | 0.066 | -0.067 | 0.650 |
| *Bacteroides_nordii* | -0.196 | 0.065 | -0.041 | 0.792 |
| *Enterobacter_cloacae* | -0.197 | 0.064 | -0.005 | 0.977 |
| *Bacteroides_clarus* | -0.201 | 0.057 | -0.056 | 0.708 |
| *Clostridium_clostridioforme* | -0.202 | 0.055 | 0.092 | 0.500 |
| *Bacteroides_finegoldii* | -0.204 | 0.052 | -0.151 | 0.191 |
| *Megamonas_rupellensis* | -0.206 | 0.049 | -0.059 | 0.692 |
| *Veillonella_atypica* | -0.206 | 0.049 | 0.017 | 0.914 |
| *Bacteroides_intestinalis* | -0.209 | 0.045 | -0.119 | 0.340 |
| *Megamonas_funiformis* | -0.211 | 0.042 | 0.050 | 0.742 |
| *Megasphaera_sp.* | -0.212 | 0.042 | -0.037 | 0.811 |
| *Lachnospiraceae_bacterium_2_1_58FAA* | -0.212 | 0.042 | -0.062 | 0.680 |
| *Megamonas_sp.* | -0.216 | 0.036 | -0.017 | 0.917 |
| *Bacteroides_cellulosilyticus* | -0.217 | 0.035 | -0.107 | 0.411 |
| *Bacteroides_fragilis* | -0.222 | 0.031 | -0.098 | 0.462 |
| *Acidaminococcus_intestini* | -0.222 | 0.031 | 0.048 | 0.752 |
| *Parabacteroides_sp.* | -0.228 | 0.025 | -0.203 | 0.053 |
| *Coprobacillus_sp.* | -0.233 | 0.021 | -0.058 | 0.698 |
| *Megamonas_hypermegale* | -0.235 | 0.020 | 0.023 | 0.889 |
| *Klebsiella_pneumoniae* | -0.237 | 0.019 | -0.021 | 0.896 |
| *Megasphaera_micronuciformis* | -0.254 | 0.010 | 0.051 | 0.736 |
| *Hungatella_hathewayi* | -0.256 | 0.009 | -0.006 | 0.968 |
| *Sutterella_wadsworthensis* | -0.260 | 0.008 | -0.193 | 0.071 |
| *Clostridium_symbiosum* | -0.268 | 0.006 | -0.020 | 0.900 |
| *Bacteroides_caccae* | -0.272 | 0.005 | -0.143 | 0.225 |
| *Flavonifractor_plautii* | -0.277 | 0.004 | -0.025 | 0.878 |
| *Clostridium_bolteae* | -0.282 | 0.003 | -0.002 | 0.987 |
| *Fusobacterium_ulcerans* | -0.284 | 0.003 | 0.097 | 0.470 |
| *Bacteroides_dorei* | -0.287 | 0.003 | -0.078 | 0.583 |
| *Parabacteroides_merdae* | -0.291 | 0.002 | 0.047 | 0.756 |
| *Veillonella_parvula* | -0.294 | 0.002 | -0.035 | 0.825 |
| *Bacteroides_xylanisolvens* | -0.302 | 0.001 | -0.118 | 0.349 |
| *Bilophila_sp.* | -0.304 | 0.001 | -0.203 | 0.054 |
| *Bilophila_wadsworthia* | -0.306 | 0.001 | -0.060 | 0.687 |
| *Parabacteroides_goldsteinii* | -0.309 | 0.001 | -0.057 | 0.702 |
| *Parabacteroides_distasonis* | -0.322 | 0.001 | -0.110 | 0.392 |
| *Bacteroides_stercoris* | -0.327 | <0.001 | -0.152 | 0.187 |
| *Clostridiales_bacterium_1_7_47FAA* | -0.328 | <0.001 | 0.022 | 0.893 |
| *Streptococcus_australis* | -0.330 | <0.001 | -0.131 | 0.277 |
| *Veillonella_sp.* | -0.337 | <0.001 | -0.108 | 0.405 |
| *Bacteroides_ovatus* | -0.360 | <0.001 | -0.170 | 0.126 |
| *Bacteroides_vulgatus* | -0.367 | <0.001 | -0.054 | 0.721 |
| *Clostridium_citroniae* | -0.379 | <0.001 | -0.096 | 0.472 |
| *Haemophilus_parainfluenzae* | -0.401 | <0.001 | -0.043 | 0.781 |
| *Bacteroides_thetaiotaomicron* | -0.433 | <0.001 | -0.033 | 0.835 |
