# Supplementary figures and images for: Metagenomic analysis of the interaction between the gut microbiota and colorectal cancer: a paired-sample study based on the GMrepo database
Source: Gut Pathog. 2022 Dec 23;14:48. doi: 10.1186/s13099-022-00527-8 (PMC9784093; doi:10.1186/s13099-022-00527-8)

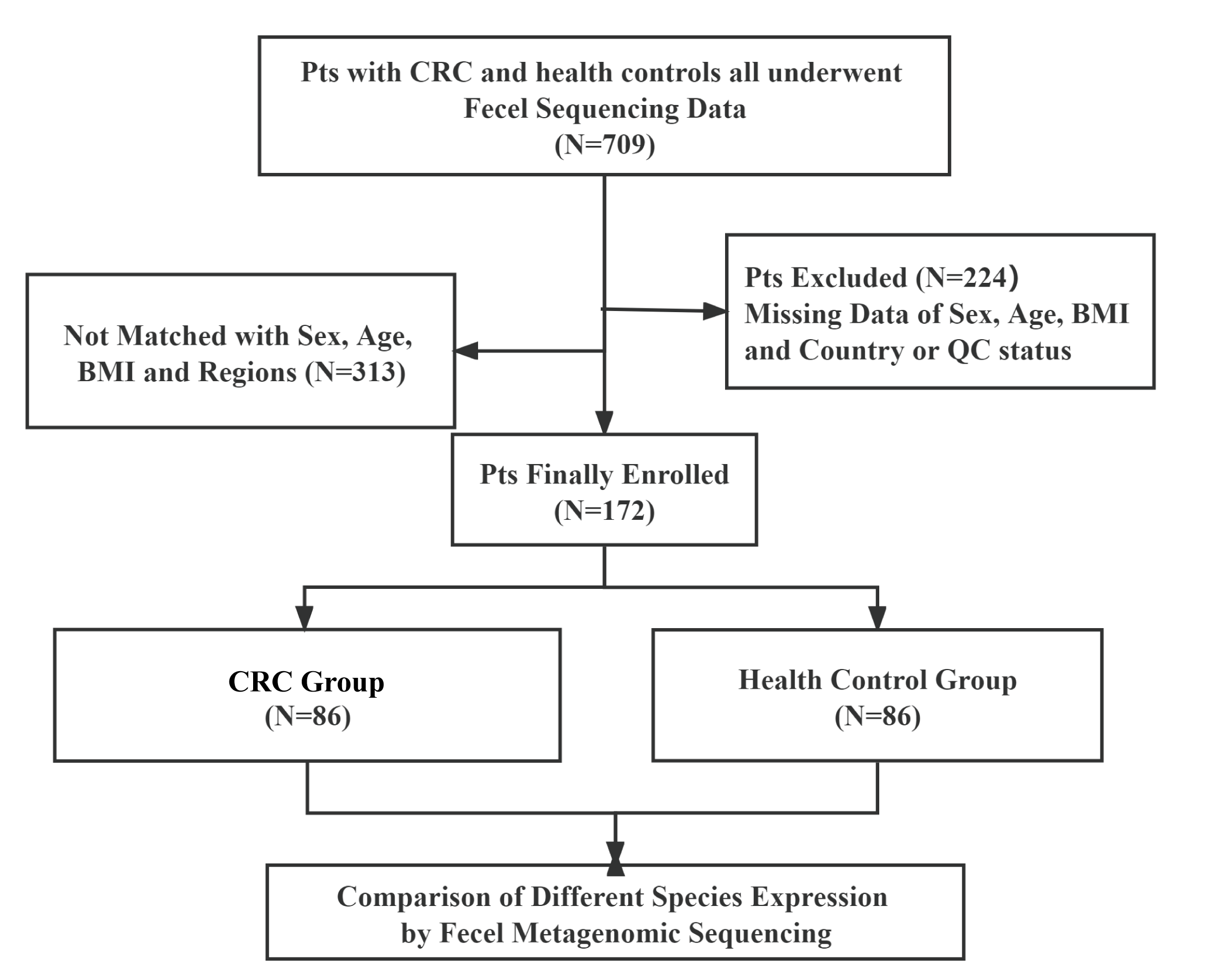

Supplement: Supplementary file 2 — Additional file 2: Figure S1. The flow chart of the study design. [file 13099_2022_527_MOESM2_ESM.tif]

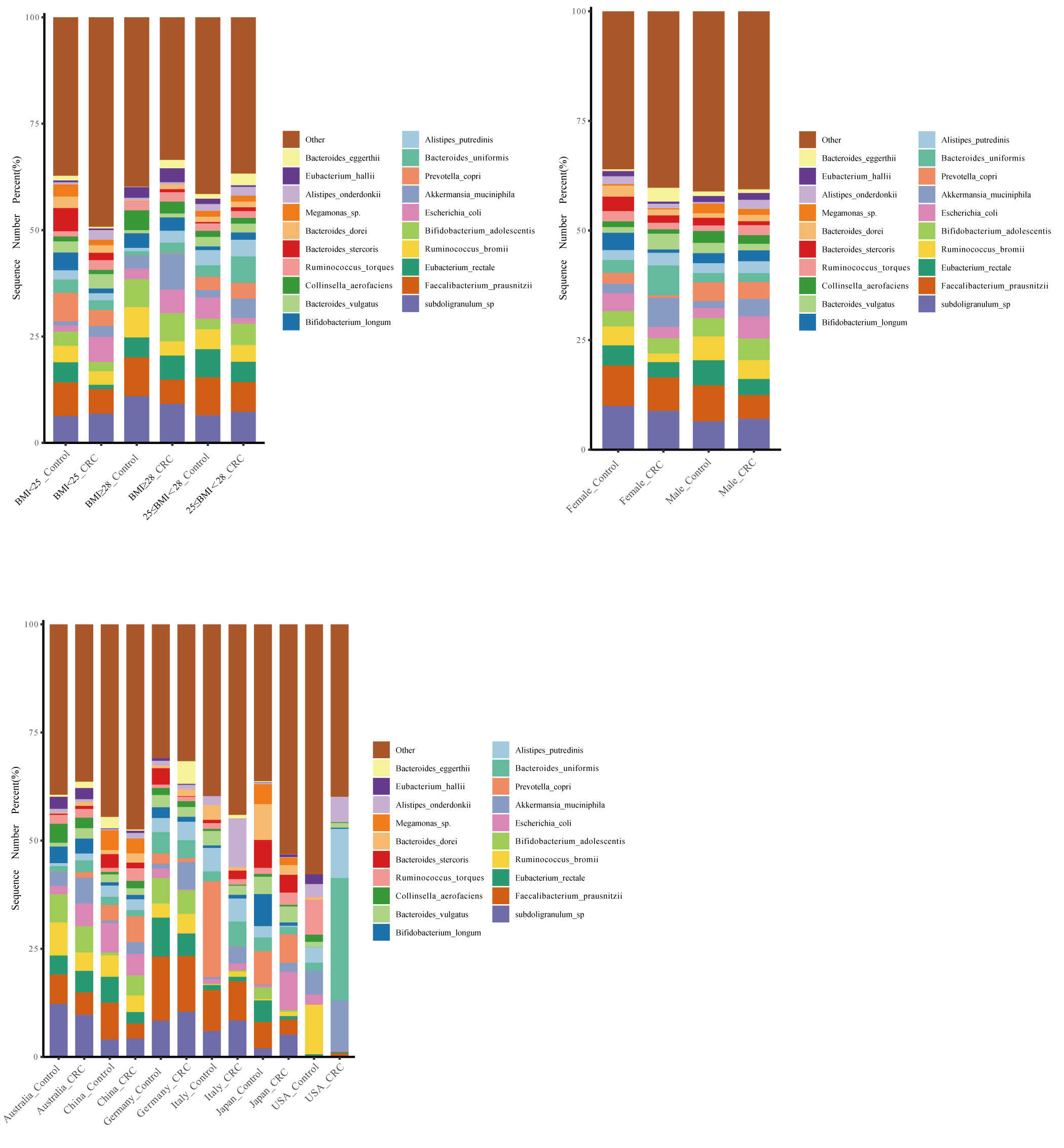

Supplement: Supplementary file 3 — Additional file 3: Figure S2. The 100% stacked column chart of relative abundance of the top 20 dominant species in CRC patients and healthy controls based on sub-BMI (A), sex (B), and region (C). The X axis represents different subgroups. The value of each species percentage in the Y-axis represents the mean of relative abundance from each subgroup. The relative abundance represents the percentage of each species made of the organism per sample. [file 13099_2022_527_MOESM3_ESM.tif]

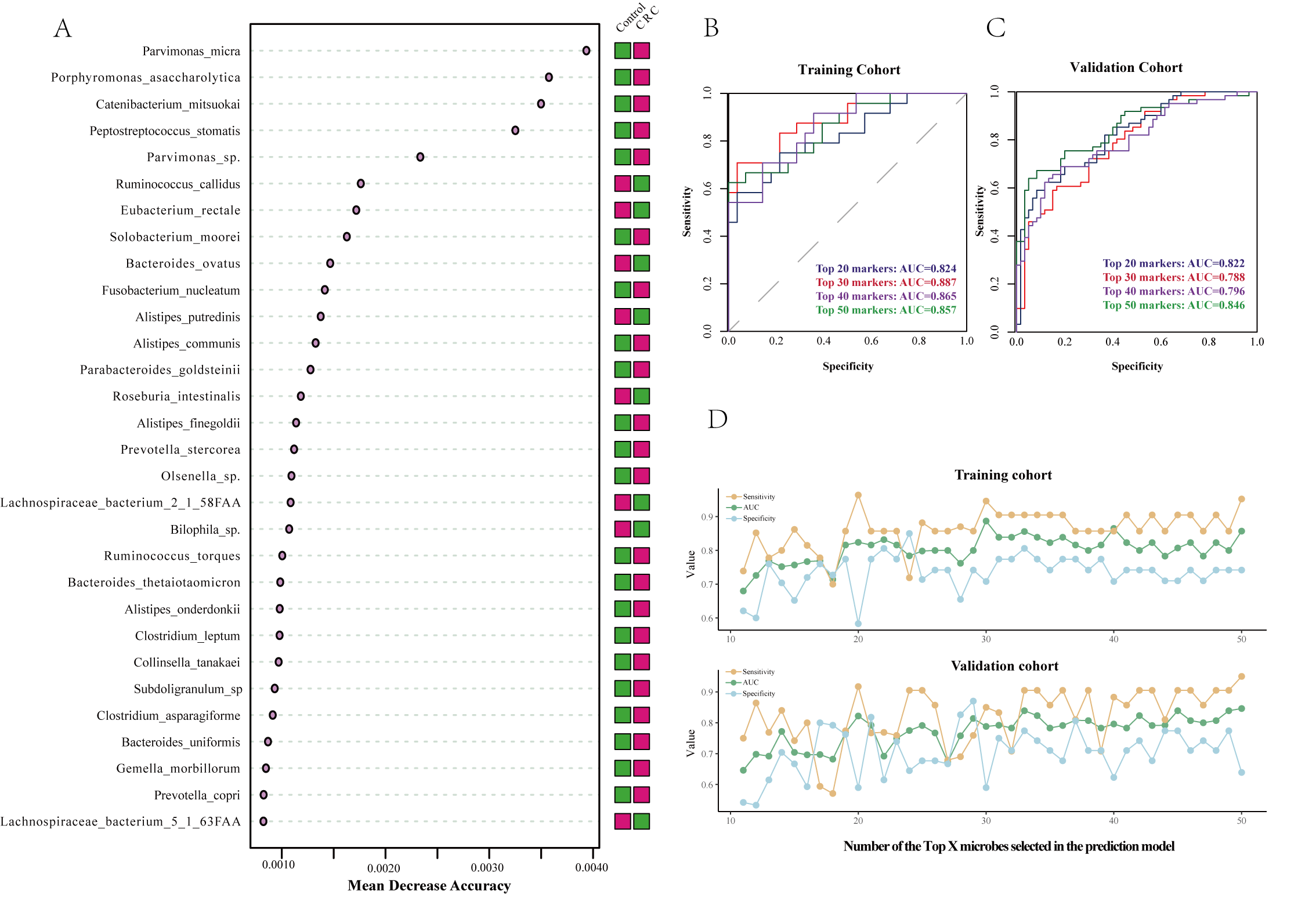

Supplement: Supplementary file 4 — Additional file 4: Figure S3. A: Random forest model of the 30 representative microbial biomarkers to predict CRC based on their mean decrease scores of the optimal model performance. The red square on the right side of each species represents the enrichment of this species in CRC groups, whereas the green square represents the enrichment of this species in controls. B-C: The area under the curve (AUC) of different models. The training and validation cohort is a 7:3 split of original data. Different AUC indexes by integrating different numbers of taxa with the highest model-building importance and lowest inner subcategory bias. D: The performance of prediction models by integrating different numbers of microbe species from the top 11 to 50 microbes. The X-axis represents the number of variables in each prediction model from the top 11 to the top 50 microbes. The Y-axis represents the value of AUC (the green curves), sensitivity (the yellow curves), and specificity (the blue curves) of each cohort. [file 13099_2022_527_MOESM4_ESM.tif]

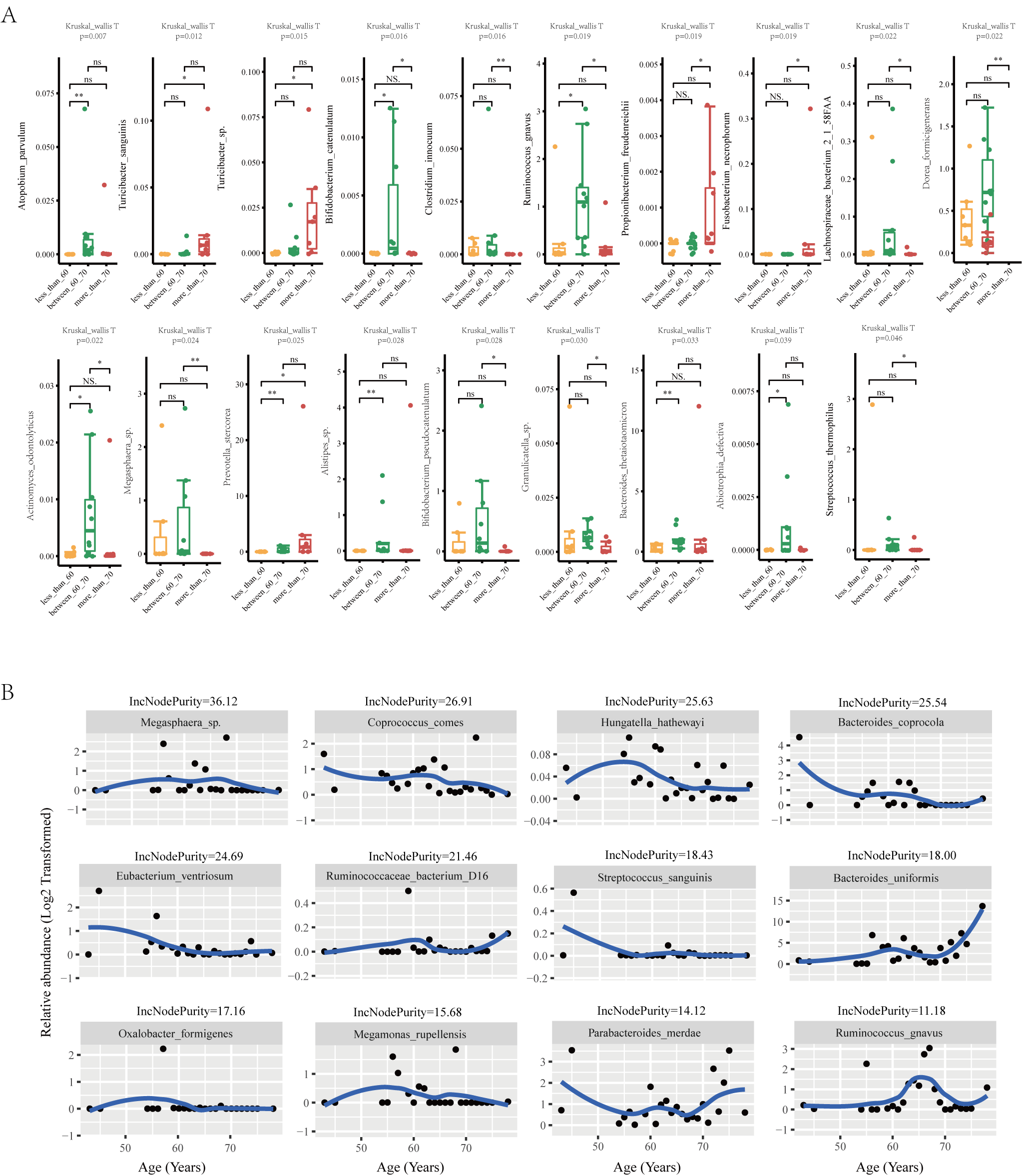

Supplement: Supplementary file 5 — Additional file 5: Figure S4. A: Species with significantly difference (Kruskal wallis Test, p<0.05) in distributions among the three sub-age groups. The boxplot displays the median of relative abundances (%) with their interquartile range. The upper and lower edge of the box represents the maximum and minimum relative abundance in each microbe, respectively. Relative abundance (%) means the percentage of a microbial species composed of the organism. The pair-wise comparisons within the subgroups were calculated using the Wilcoxon Mann-Whitney. p-value＜0.05 was considered statistical significance. (*, **, *** for p-values < 0.01, 0.005 and 0.001, respectively). B: The top 12 important species significantly associated with age in CRC patients (identified by the random forest algorithm). The Y-axis represents the relative abundance (Log2 transformed) of each species. The X-axis represents age as continuous variable. The relative abundance represents the percentage of each species made of the organism per sample. The Increased Node Impurity Index (IncNodePurity) was listed on the top of each species. Microbes with higher values of the IncNodePurity are considered to have higher association with age. [file 13099_2022_527_MOESM5_ESM.tif]

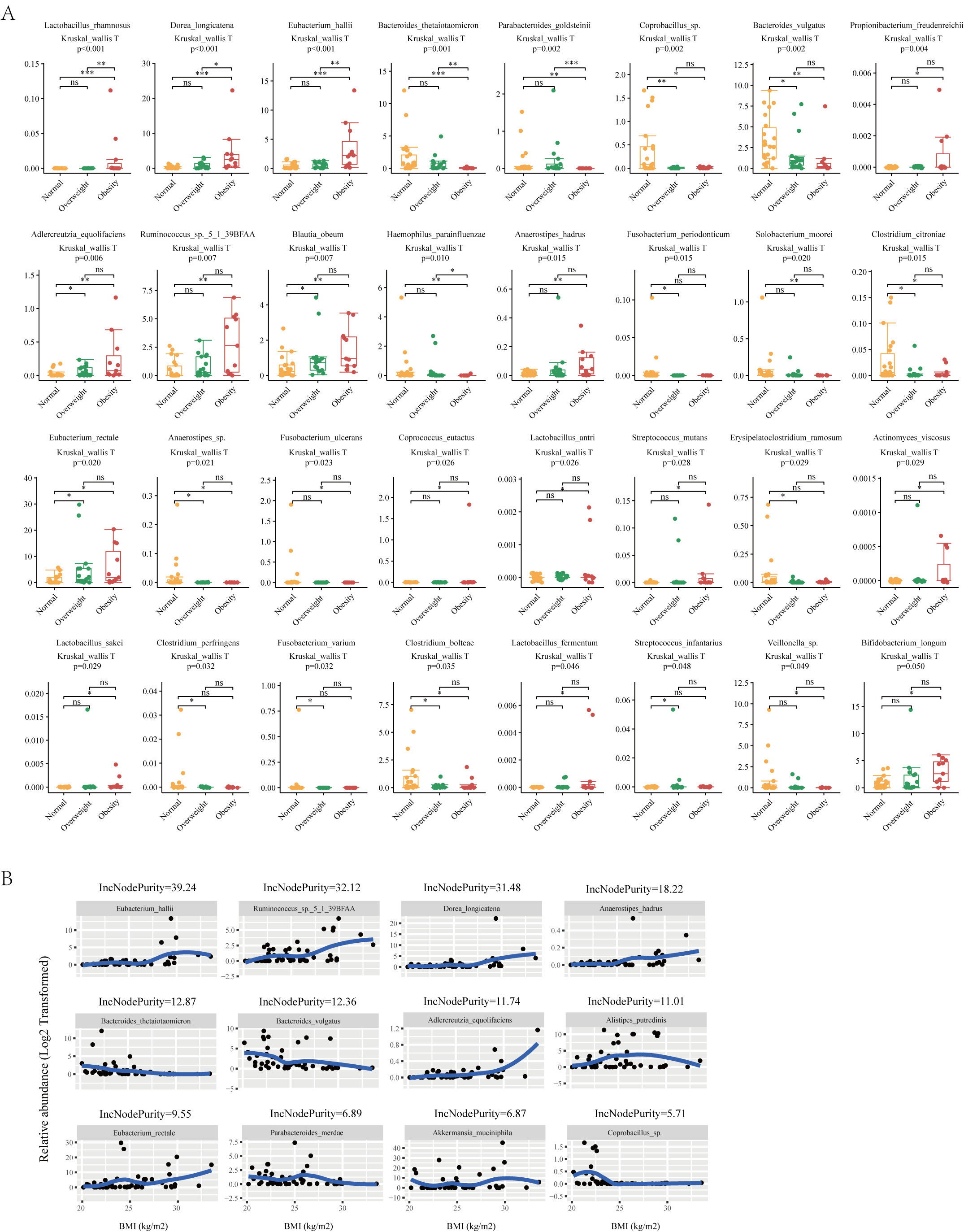

Supplement: Supplementary file 6 — Additional file 6: Figure S5. A: Species with significantly difference (Kruskal wallis Test, p<0.05) in distributions among the three sub-BMI groups. The boxplot displays the median of relative abundances (%) with their interquartile range. The upper and lower edge of the box represents the maximum and minimum relative abundance in each microbe, respectively. Relative abundance (%) means the percentage of a microbial species composed of the organism. The pair-wise comparisons within the subgroups were calculated using the Wilcoxon Mann-Whitney. p-value＜0.05 was considered statistical significance. (*, **, *** for p-values < 0.01, 0.005 and 0.001, respectively). B: The top 12 important species significantly associated with age in CRC patients (identified by the random forest algorithm). The Y-axis represents the relative abundance (Log2 transformed) of each species. The X-axis represents age as continuous variable. The relative abundance represents the percentage of each species made of the organism per sample. The Increased Node Impurity Index (IncNodePurity) was listed on the top of each species. Microbes with higher values of the IncNodePurity are considered to have higher association with BMI. [file 13099_2022_527_MOESM6_ESM.tif]
